# Influence of autozygosity on common disease risk across the phenotypic spectrum

**DOI:** 10.1016/j.cell.2023.08.028

**Published:** 2023-10-12

**Authors:** Daniel S. Malawsky, Eva van Walree, Benjamin M. Jacobs, Teng Hiang Heng, Qin Qin Huang, Ataf H. Sabir, Saadia Rahman, Saghira Malik Sharif, Ahsan Khan, Maša Umićević Mirkov, 23andMe Research Team, Genes & Health Research Team, Hiroyuki Kuwahara, Xin Gao, Fowzan S. Alkuraya, Danielle Posthuma, William G. Newman, Christopher J. Griffiths, Rohini Mathur, David A. van Heel, Sarah Finer, Jared O’Connell, Hilary C. Martin

**Affiliations:** 1 Wellcome Sanger Institute, Wellcome Genome Campus, Hinxton, UK; 2 Department of Clinical Genetics, Amsterdam UMC, University of Amsterdam, Amsterdam, the Netherlands; 3 Department of Complex Trait Genetics Center for Neurogenomics and Cognitive Research, Amsterdam Neuroscience, VU Amsterdam, Amsterdam, the Netherlands; 4 Blizard Institute, Barts and the London School of Medicine and Dentistry, Queen Mary University of London, London, UK; 5 Wolfson Institute of Population Health, Queen Mary University of London, London, UK; 6 West Midlands Regional Clinical Genetics Unit, Birmingham Women’s and Children’s NHS FT, Birmingham, UK; 7 Institute of Cancer and Genomics, College of Medical and Dental Sciences, University of Birmingham, Birmingham, UK; 8 Queen Square Institute of Neurology, University College London, London, UK; 9 Yorkshire Regional Genetics Service, Leeds Teaching Hospitals NHS Trust, Leeds, UK; 10 Waltham Forest Council, Waltham Forest Town Hall, Forest Road, Walthamstow E17 4JF, UK; 11 Congenica Limited, BioData Innovation Centre, Wellcome Genome Campus, Hinxton, UK; 12 King Abdullah University of Science and Technology (KAUST), Computational Bioscience Research Center (CBRC), Thuwal 23955, Saudi Arabia; 13 Department of Translational Genomics, Center for Genomic Medicine, King Faisal Specialist Hospital and Research Center, Riyadh, Saudi Arabia; 14 Division of Evolution, Infection and Genomics, Faculty of Biology, Medicine and Human Sciences, University of Manchester, Manchester M13 9PL, UK; 15 Manchester Centre for Genomic Medicine, Manchester University NHS Foundation Trust, Manchester M13 9WL, UK; 16 MRC and Asthma UK Centre in Allergic Mechanisms of Asthma, King’s College London, London, UK; 17 23andMe, Inc., Sunnyvale, CA, USA

**Keywords:** consanguinity, autozygosity, common diseases, recessive, medical genetics, diverse cohorts

## Abstract

Autozygosity is associated with rare Mendelian disorders and clinically relevant quantitative traits. We investigated associations between the fraction of the genome in runs of homozygosity (F_ROH_) and common diseases in Genes & Health (n = 23,978 British South Asians), UK Biobank (n = 397,184), and 23andMe. We show that restricting analysis to offspring of first cousins is an effective way of reducing confounding due to social/environmental correlates of F_ROH_. Within this group in G&H+UK Biobank, we found experiment-wide significant associations between F_ROH_ and twelve common diseases. We replicated associations with type 2 diabetes (T2D) and post-traumatic stress disorder via within-sibling analysis in 23andMe (median n = 480,282). We estimated that autozygosity due to consanguinity accounts for 5%–18% of T2D cases among British Pakistanis. Our work highlights the possibility of widespread non-additive genetic effects on common diseases and has important implications for global populations with high rates of consanguinity.

## Highlights

• Robust method to reduce confounding in autozygosity-phenotype association studies
• Higher autozygosity associated with increased risk for common diseases such as T2D
• Replication of findings including a within-sibling analysis
• Consanguinity explains ∼10% of T2D cases in British Pakistanis

## Graphical abstract

**Figure:**
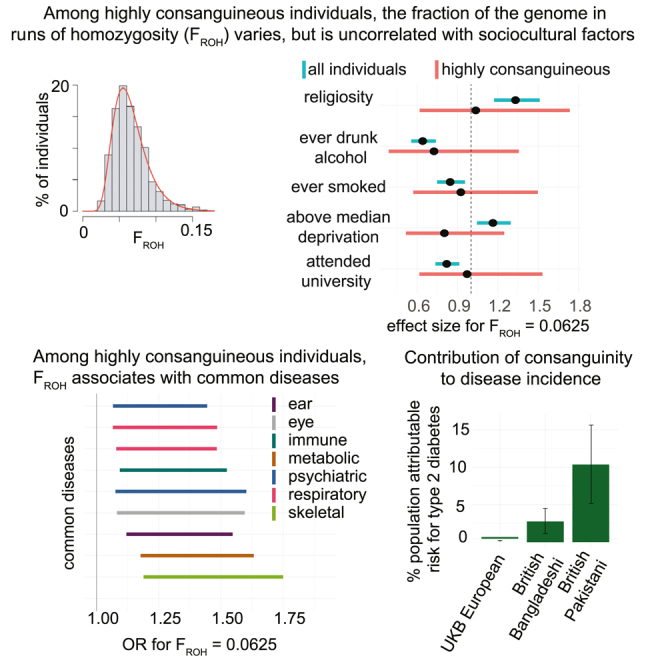

## Teaser

Autozygosity resulting from consanguinity is causally associated with several complex diseases, including type 2 diabetes.

## Introduction

The prevalence of consanguinity, unions between related individuals, differs around the world, being relatively low in Europe and higher in South Asia and the Middle East.^1^^,^^2^ It often co-occurs with endogamy—unions between individuals from the same clan or social group.^3^^,^^4^^,^^5^ These practices increase the rates of autozygosity, i.e., stretches of homozygosity in the genome that are identical by descent.^6^ Autozygosity is known to increase the risk of rare congenital anomalies and recessive Mendelian disorders,^7^^,^^8^ and it has been associated with various other phenotypic outcomes, such as decreased height, fertility, and self-reported overall health,^9^^,^^10^ as well as increased risk for complex diseases such as Alzheimer’s disease^11^ and coronary artery disease (CAD).^12^ Notably, the prevalence of CAD and other complex diseases such as type 2 diabetes (T2D) is significantly higher in British South Asian individuals compared with White British individuals.^13^ Although this is undoubtedly partly due to social and environmental factors^13^^,^^14^ as well as differential additive genetic susceptibility toward T2D at certain loci in South Asians compared with White Europeans,^15^ it is unclear whether higher rates of autozygosity could also contribute.

One mechanistic explanation for the association between autozygosity and certain traits and diseases is that autozygosity increases the chance of harboring rare homozygous genotypes at damaging recessive variants, which are less effectively removed from the population by negative selection than dominantly acting variants.^16^ However, other potential explanations exist, such as the heterozygote advantage hypothesis, whereby heterozygosity for certain common variants leads to fitness advantages^16^ or that the increased variance in additive genetic liability toward binary traits induces associations with autozygosity in the absence of non-additive effects.^17^

A challenging problem in assessing the relationship between autozygosity and phenotypes is that associations may be confounded by both population structure and the social circumstances in which consanguinity and endogamy are practiced. For example, attempted replication of a previously detected association with schizophrenia^18^ failed in reasonably powered cohorts,^19^^,^^20^ suggesting potential confounding. In another example, it has been shown that a negative association in the Netherlands between depression and the fraction of the genome in runs of homozygosity (ROHs) (F_ROH_, a measure of autozygosity) was confounded by religious assortative mating, whereby religious individuals had higher F_ROH_ due to stricter endogamy.^21^ Thus, the environmental and social factors that correlate with having related parents may produce spurious associations between autozygosity and disease phenotypes. However, experimental studies in nonhuman organisms that are free of social and environmental confounding confirm the effects of autozygosity on several phenotypes,^16^^,^^22^^,^^23^^,^^24^^,^^25^^,^^26^ suggesting that the observations in humans may be at least partially of genetic origin.

Here, we describe the patterns of consanguinity and examine the effect of autozygosity on disease risk across the phenotypic spectrum in two cohorts: the Genes & Health (G&H) cohort, a population-based study of self-identified British Bangladeshi and British Pakistani individuals, and UK Biobank (UKB) individuals genetically inferred to have majority European and South Asian ancestries. We show that subsetting association analyses to highly consanguineous individuals better controls for social and environmental confounding. With this approach, we find significant associations between autozygosity and various diseases, several of which we replicate using a different method in a within-sibling analysis conducted in the 23andMe cohort. Via simulations, we show that these observed associations most likely stem from non-additive genetic effects. Our study quantifies the effect of autozygosity across the disease phenotypic spectrum using a robust approach that addresses confounding and highlights the possibility of widespread non-additive effects across diseases.

Since consanguinity is a sensitive topic for many communities, we have prepared a “frequently asked questions” document for a lay audience in collaboration with the Community Advisory Board from G&H, explaining the motivation for and results of our study and placing them in a wider context.

## Results

Our main analysis focuses on two cohorts, G&H and UKB, both with electronic health record (EHR) data from primary and secondary care provided by the National Health Service (NHS) in England. G&H is a community-based cohort of individuals self-identifying as British Bangladeshi (65%) and Pakistani (35%), recruited in London, Manchester, and Bradford, UK (n = 44,190 with genetic and EHR data at the time of analysis). The dataset is reasonably representative of the background population, albeit likely with some over-sampling of individuals with chronic diseases since much of the recruitment was conducted in a primary care setting.^27^ We additionally analyzed individuals from the UKB. We removed individuals for whom EHR data linkage was unavailable, and one of each pair of individuals was inferred to be third-degree relatives or closer.

We began by classifying G&H and UKB individuals into genetically inferred ancestry (GIA) groups, as described in the section “Inference of genetic ancestry” in STAR Methods. The rationale for this was two-fold. Firstly, we were interested in exploring patterns of consanguinity that might differ between people from different genetic backgrounds. Secondly, we also wanted to explore the effects of autozygosity on disease while ensuring that these were not confounded by environmental or cultural factors that might be correlated with consanguinity and with GIA. We recognize that these GIA groups do not capture the full genetic diversity of human populations and that individuals with a particular national identity, such as “Pakistani” or “Bangladeshi,” may have varying ancestries. We analyzed 23,978 G&H individuals, of which 8,122 and 15,856 had majority Pakistani and Bangladeshi GIA, respectively (referred to henceforth as “British Pakistanis” and “British Bangladeshis”), 387,531 UKB individuals with majority European GIA (UKB EUR), and 9,653 UKB individuals with majority South Asian GIA (UKB SAS). See Table 1 for descriptive statistics of the cohorts.

**Table 1:** Descriptive statistics of unrelated individuals in the G&H and UKB cohorts

	G&H (n = 23,978)	UKB EUR (n = 387,531)	UKB SAS (n = 9,653)
% male	47%	46%	54%
Age (years)—mean (SD)	44.9 (13.1)	56.7 (8.0)	53.4 (8.5)
Self-reported ethnic background	65% Bangladeshi, 35% Pakistani	94% Great Britain, 6% other European	60% Indian, 21% Pakistani, 4% Bangladeshi, 15% other South Asian
F_ROH_ mean (SD)	0.0178 (0.025)	0.0037 (0.0050)	0.013 (0.022)
# “highly consanguineous”	4,034 (16.8%)	977 (0.25%)	754 (7.8%)

F_ROH_ is the fraction of the genome in runs of homozygosity. The bottom row gives the number of individuals inferred to be offspring of first cousin/avuncular unions included in the “highly consanguineous” analyses. SD, standard deviation.

### Consanguinity patterns in G&H and UKB

**Figure 1:**
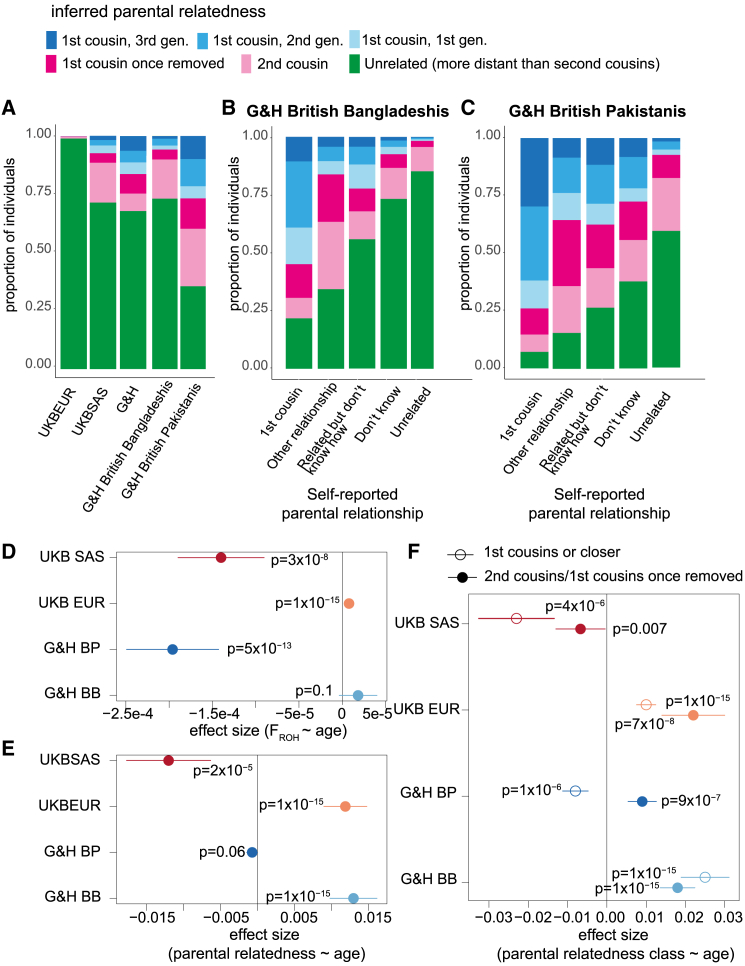
Patterns of parental relatedness (PR) in G&H and UKB (A) Stacked bar plots showing genetically inferred PR for the indicated groups. The inferred classes of PR include up to three generations of first cousin marriages, first cousins once removed, and second cousins or unrelated. (B and C) Stacked bar plot showing genetically inferred PR for G&H British Bangladeshi and British Pakistani individuals, respectively, stratified by self-reported PR. (D) Effect sizes of age on F_ROH_, inferred from linear regression, in the indicated groups. (E) Effect sizes of age on being genetically inferred offspring of second cousins or closer, from logistic regression. (F) Effect sizes of age having the indicated class of PR, inferred from multinomial logistic regression. Lines indicated 95% confidence intervals. BB, British Bangladeshi; BP, British Pakistani. See also Figure S1.

Given that G&H has high self-reported rates of consanguinity^27^ (9% in British Bangladeshi individuals and 36% in British Pakistani individuals), we first sought to genetically characterize consanguinity patterns in the cohort and compare them with UKB. We applied a method we previously developed to infer an individual’s parental relatedness (PR) based on the distribution of ROHs in their genome.^2^ The method infers ten classes of PR, some involving multiple generations of consanguinity (STAR Methods). Rates of consanguinity (offspring of second cousins or closer) were very low in UKB EUR (2%) and higher in UKB SAS and G&H (29% and 33%, respectively) (Figure 1A). In concordance with previous findings in G&H based on F_ROH_ distribution,^27^ self-reporting of PR was imperfect (Figures 1B and 1C).

**Figure S1:**
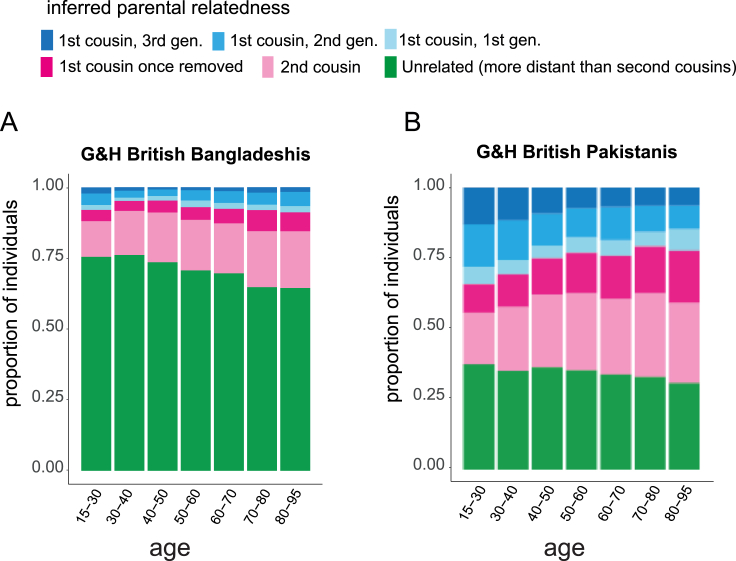
Stacked bar plots showing inferred parental relatedness by age bin in G&H, related to Figure 1 (A) British Bangladeshi individuals and (B) British Pakistani individuals. The inferred categories of parental relatedness include up to three generations of first cousin marriages, first cousins once removed, second cousins or unrelated.

Next, we explored whether the rate of consanguinity has been changing over time (Figures 1D–1F). We replicated a recent finding^28^ that in UKB EUR, F_ROH_ significantly increases with age (Figure 1D). In contrast, F_ROH_ significantly decreases with age in G&H British Pakistani individuals but shows no significant association in G&H British Bangladeshi individuals (Figure 1D). In UKB EUR and G&H British Bangladeshi individuals, age was significantly positively associated with rates of both first cousin or closer PR and of first cousins once removed/second cousin PR (Figure 1F). In G&H British Pakistani individuals, although there is no significant overall change in the rate of PR (i.e., second cousin or closer) with age (Figure 1E), we see significant and opposing age effects for different classes of PR (Figure 1F). We note that although these trends are highly significant, the changes are relatively modest; for example, 23% of British Pakistani individuals aged 70–80 years were inferred to be offspring of first cousins or closer, compared with 38% of those aged 15–30 years (Figure S1).

### Associations between autozygosity and common confounders

**Figure 2:**
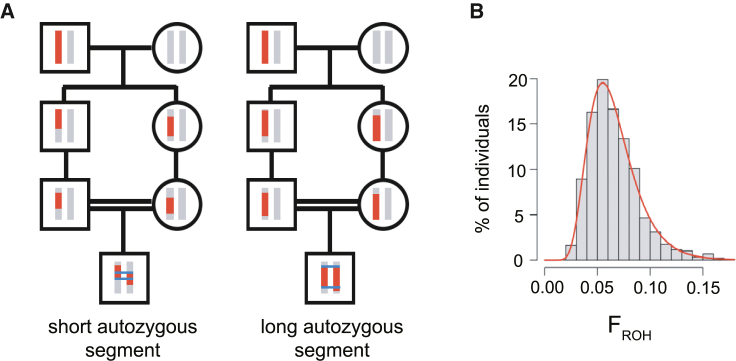
Variability in autozygosity due to stochastic recombination and Mendelian segregation events among individuals with parents who are first cousins (A) Figure illustrating, using just one chromosome, how autozygosity can vary substantially between individuals who are offspring of first cousins. Two offspring of independent first cousin unions have inherited different ROHs of different lengths on one chromosome due to stochastic recombination and Mendelian segregation events. (B) This leads to the variation in genome-wide F_ROH_ shown in (B) for G&H individuals inferred to have parents who are first cousins. The red line in (B) indicates the best fit of a lognormal distribution, which was used for power calculations.

We then examined associations between F_ROH_ and phenotypes in G&H and UKB, considering two sets of individuals within each cohort; we carried out one version of the analyses using all individuals (full cohort) and one using only individuals who are inferred to be offspring of first cousin/avuncular unions and who have F_ROH_ < 0.18 (highly consanguineous cohort). (The cutoff of F_ROH_ < 0.18 was chosen as it is the midpoint between the expected F_ROH_ for individuals having avuncular versus sibling parents). The motivation for this was that we suspected that social and environmental correlates of consanguinity may confound associations between phenotypes and F_ROH_ within the full cohort, i.e., highly consanguineous individuals might have systematically different cultural, social, or environmental exposures to those whose parents are unrelated. If we restrict to individuals whose parents had the same degree of PR and control for population structure, variance in F_ROH_ is attributable to stochastic recombination events and Mendelian segregation (Figure 2), thus mitigating associations between F_ROH_ and environmental confounders. We excluded the small number of individuals with F_ROH_ > 0.18 whose parents may be first-degree relatives, since such unions might be associated with extreme environmental confounders.

**Figure 3:**
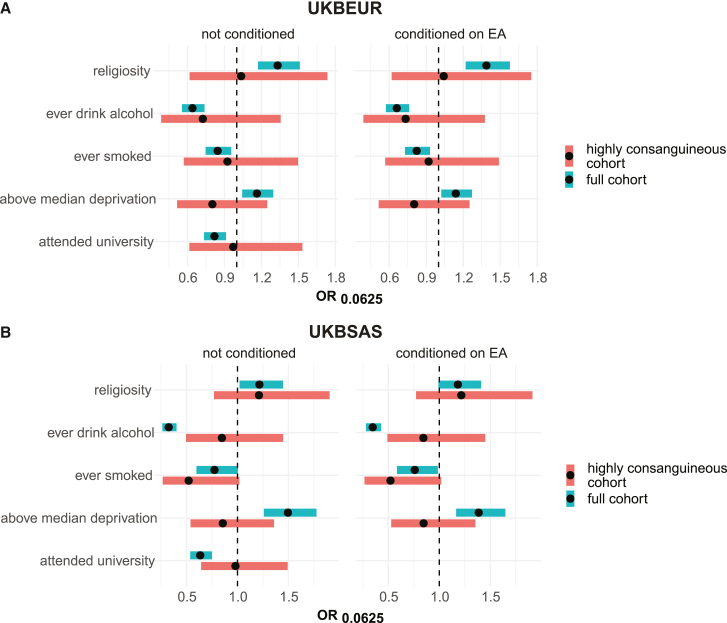
Associations between F_ROH_ and potential confounders in UKB Associations between F_ROH_ and potential confounders with (right) and without (left) conditioning on educational attainment (EA) in (A) UKB EUR and (B) UKB SAS. Forest plot showing F_ROH_ odds ratio. OR is calculated for F_ROH_ value of 0.0625 (expected F_ROH_ for first cousin PR). Bands indicate 95% confidence intervals adjusted for multiple testing (p < 0.05/9).

To test the robustness of this approach, we first considered five traits/exposures that may confound associations with F_ROH_ in UKB EUR and UKB SAS—self-reported religiosity, having ever smoked tobacco, having ever drunk alcohol, socioeconomic status (SES) as measured by the Townsend deprivation index, and having attended university. Clark et al. previously showed that F_ROH_ negatively correlated with educational attainment (EA) and alcohol and tobacco use.^9^ We find that in the full cohort, F_ROH_ is significantly associated with all five traits assessed in UKB EUR and UKB SAS (Figure 3). However, in the highly consanguineous cohorts, we find no significant associations. Using power calculations,^29^ we find that the power to detect significant associations in the highly consanguineous cohorts using the odds ratio (OR) estimated from the full cohorts ranges from 0.72 to >0.99 with a median of 0.86, suggesting the widespread attenuation observed was unlikely to be due to the reduction in sample size when restricting to the highly consanguineous cohorts. As has been done in previous work to attempt to control for confounding,^9^^,^^10^^,^^30^ we then repeated these analyses controlling for EA (number of years in education). This made a minimal difference to our results (Figure 3, right), showing that conditioning on EA does not attenuate associations with the potential confounders we considered.

As a complementary analysis, we sought to quantify the variance in F_ROH_ explained collectively by the above confounders in addition to body mass index (BMI), dietary patterns, and exercise frequency-related variables (STAR Methods). Our goal was to quantify the degree of potential confounding and increase the power to detect confounding in aggregate. To do so, we regressed F_ROH_ on the confounders within the full and highly consanguineous cohorts and assessed the model fit. In both full cohorts (UKB EUR and UKB SAS), the models are highly significant, with adjusted-R^2^ estimates greater than 0.01 (Table S1). However, in the highly consanguineous cohorts, the model was not significant (p > 0.3), with adjusted R^2^ < 0.001. Notably, for UKB SAS, we found we have >80% statistical power to detect an R^2^ half as large as that detected in the full cohort (Table S1), suggesting that the lack of significant model fit is not simply due to lower power in the highly consanguineous cohort compared with the full cohort.

### Associations between autozygosity and disease

Having demonstrated that focusing on highly consanguineous individuals reduces confounding with risk factors for ill health, we then assessed associations between F_ROH_ and diseases in this subset of individuals, meta-analyzing G&H and UKB. We first considered height as a positive control,^9^ noting a significant association between it and F_ROH_ in the highly consanguineous cohort (β = −0.93 cm, p = 1.9 × 10^−5^ for F_ROH_ = 0.0625) that was not significantly different (p = 0.54, two-sample t test) from the effect in the full cohort (β = −1.07 cm, p < 10^−10^) (Table S2). Then, to define the disease phenotypes, we used the first-occurrence three-letter International Classification of Diseases (ICD10) codes in UKB and generated phenotypes in G&H by mapping diagnostic codes from primary and secondary care EHRs using the methods defined in UKB (STAR Methods). We considered the sixty-one diseases with at least a 5% case prevalence in the G&H highly consanguineous cohort, since this was the largest sample (n = 4,034 versus n = 977 and n = 754 for UKB EUR and UKB SAS, respectively).

**Figure 4:**
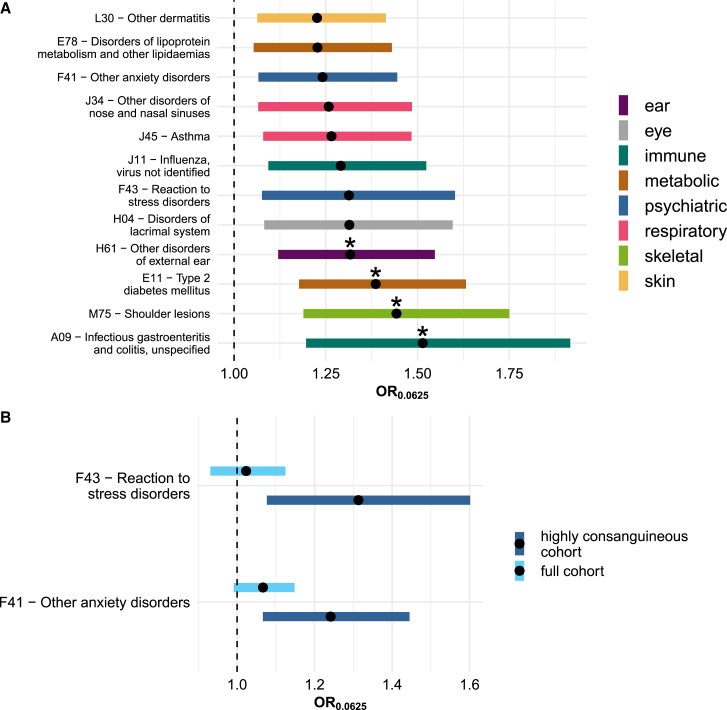
Associations between F_ROH_ and disorders significant after 5% FDR correction in the meta-analysis of highly consanguineous cohorts from G&H and UKB (A) shows all significant disorders, and (B) highlights two psychiatric disorders that showed significant associations in the meta-analysis of highly consanguineous cohorts but not of full cohorts. Forest plot showing F_ROH_ odds ratio (OR). OR is calculated for F_ROH_ value of 0.0625 (expected F_ROH_ for first cousin PR). Bands indicate 95% confidence intervals, asterisks indicate traits that pass Bonferroni correction (p < 0.05/61), and colors indicate disorder categories. See also Figures S2, S3, and S5.

After 5% false discovery rate (FDR) correction, we found twelve associations, with four associations passing Bonferroni correction (p < 0.05/61) in the meta-analysis of the highly consanguineous cohorts (Figure 4A; Table S3). The disorders span several organ systems, including metabolic, psychiatric, ear, eye, immune, and respiratory disorders. When conducting the same analysis in the full cohorts, thirty and thirteen diseases passed FDR and Bonferroni corrections, respectively (Table S3). The highly consanguineous and full cohort analyses share ten significant associations at FDR < 5%, with the two psychiatric traits being unique to the former (Figure 4B). One of the most significant associations seen in both the highly consanguineous and full cohort analyses was with T2D (highly consanguineous cohort: OR = 1.39 for F_ROH_ = 0.0625, 95% confidence interval [CI] = [1.17,1.63], p = 8.5 × 10^−5^). We replicated this in a set of individuals inferred to be offspring of first cousin/avuncular unions (n = 1,476) from a cohort of Saudi Arabs, with effect size consistent with what we saw in G&H+UKB (OR = 1.31 for F_ROH_ = 0.0625, 95% CI = [1.06, 1.62], p = 0.012).

**Figure S2:**
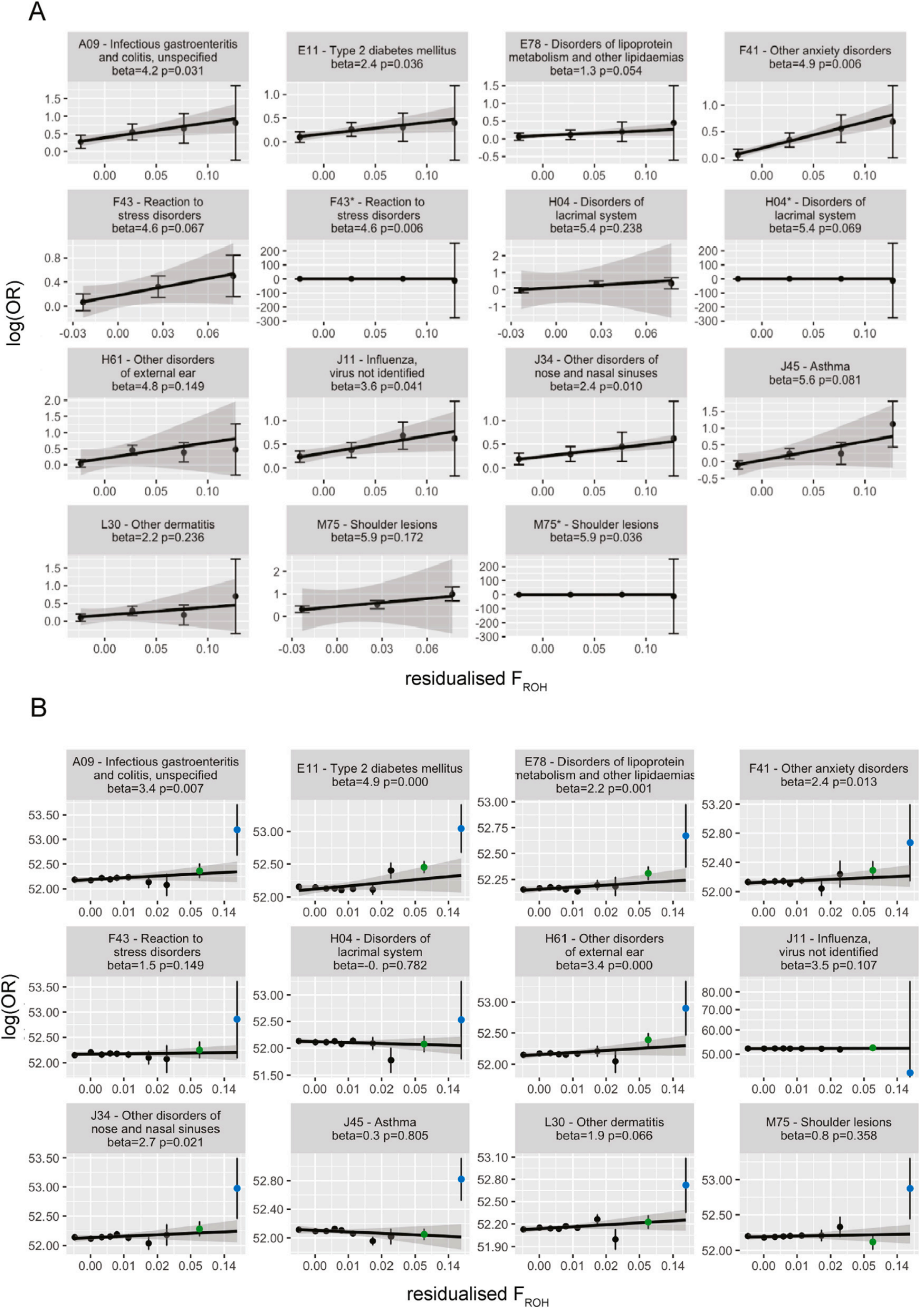
Log(OR) increase in disease risk across residualized F_ROH_ bins, related to Figure 4 and Table S3 Log(OR) increase in disease risk across residualized F_ROH_ bins by quintiles in the meta-analysis of the (A) highly consanguineous cohorts and (B) full cohorts. Note that in (B), the second-to-rightmost point (green) includes >95% of the individuals who were included in the highly consanguineous analysis, and the rightmost point (light blue) represents individuals with F_ROH_ > 0.18 who were excluded from it. The log(OR) is expressed with respect to the lowest quintile of residualized F_ROH_ values. Error bars reflect standard error (SE), and lines reflect a linear regression of log(OR) on residualized F_ROH_ values, with shading representing the SE of the slope. For some traits in (A), the log(OR) SEs were >200 for the last quintile. For these traits, we plotted them twice, once excluding the last quintile estimate and once including the estimate, designated with a ^∗^ following the three-letter-code. Effect sizes (beta) and p values are from an inverse-variance-weighted linear regression of the log(OR) on the residualized F_ROH_ quintiles.

**Figure S3:**
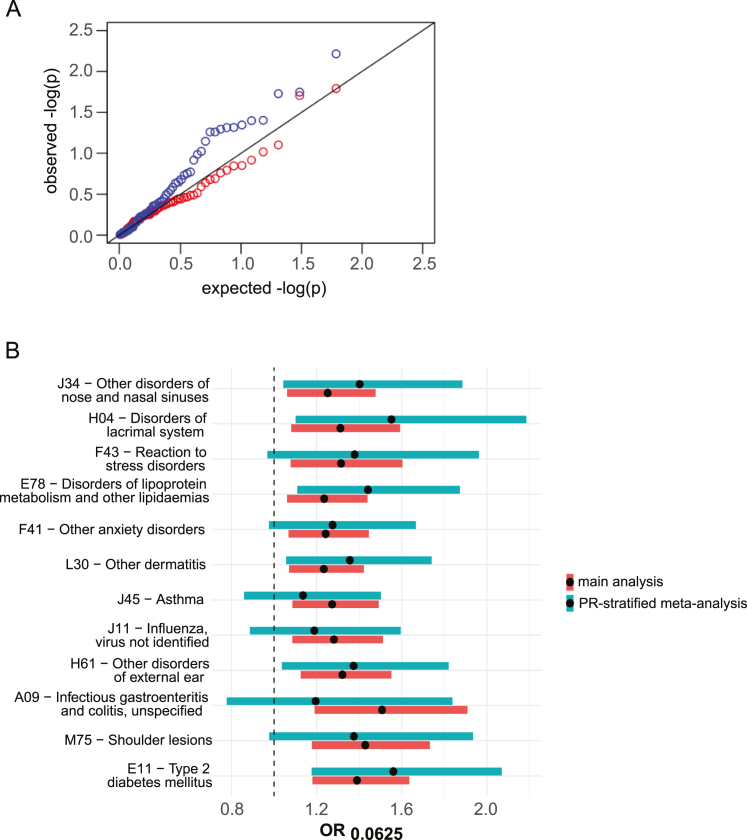
Assessing robustness of F_ROH_-phenotype associations in the highly consanguineous cohorts, related to Figure 4 and Table S3 (A) Quantile-quantile plot of p values from a Cochran’s Q test for heterogeneity for all 61 diseases tested across G&H, UKB EUR, and UKB SAS, using the full cohorts (blue) and the highly consanguineous cohorts (red). The black line is y = x. (B) Comparing the effect size estimates for the highly consanguineous analysis (main analysis; red) to those from an analysis in which we ran regressions separately on individuals descended from one, two, or three generations of first cousin marriage, meta-analyzed these results per cohort, and then conducted a cross-cohort meta-analysis (PR-stratified meta-analysis; teal).

We assessed whether the effect of F_ROH_ varied linearly with respect to the log-odds within the highly consanguineous cohorts using binned F_ROH_ values to ensure model assumptions were met. We find that the increase in log-odds for the significant traits consistently appears to be approximately linear (Figure S2A), suggesting the associations are not driven by extreme F_ROH_ values. However, a similar analysis in the full cohort highlights nonlinearities in the log-odds across the range of F_ROH_ outside the range considered in the highly consanguineous analysis (Figure S2B), suggesting uncontrolled confounding. We also observe inflation in the p values for Cochran’s Q test for heterogeneity in the meta-analysis of the full cohorts and none for the highly consanguineous cohorts (Figure S3A), suggesting that the effect size estimates are more consistent across the latter.

As an additional sensitivity analysis, we refitted the regression for the significant associations after stratifying each highly consanguineous cohort by the number of generations of inferred first cousin PR (one, two, or three). We meta-analyzed these three regressions within each of UKB EUR, UKB SAS, and G&H and then meta-analyzed these results across cohorts (“PR-stratified meta-analysis”; Figure S3B; Table S4). The rationale for doing so was to ensure that within the highly consanguineous group, the results were not confounded by social/environmental correlates of the number of generations of recent historic consanguinity. Although this analysis reduces power, several associations remained significant, and effect sizes were highly similar, with the average ratio of the effect size in the PR-stratified meta-analysis compared with that in the original highly consanguineous analysis being 1.055 (paired t test p = 0.486). We additionally refitted the regressions in the highly consanguineous UKB EUR and UKB SAS cohorts for the significant phenotypes, adding the confounders assessed in Table S1 as covariates, and found no reduction in effect size magnitude; the ratio of the effect size from regression with the additional covariates and the original regressions was 1.05 in UKB SAS and 1.10 in UKB EUR (p = 0.73 and p = 0.47, respectively, paired t test), and no phenotype had a nominally significant difference in the effect size estimates ascertained from the two regressions (Table S4).

### Within-sibling analysis of F_ROH_-phenotype associations in 23andMe

To attempt to replicate findings, we conducted a within-sibling analysis in the 23andMe cohort using self-reported phenotypes (n = 42,218–545,806 siblings, median 478,590; Table S5). This complementary approach exploits variation in F_ROH_ within nuclear families, which eliminates confounding due to population structure.^9^^,^^31^^,^^32^ Confirming the results in Figure 3, we found no significant association (p > 0.15) between F_ROH_ and having ever used tobacco or reporting being “at all religious.”

We then considered fourteen disease phenotypes that match or are similar to the three-digit ICD10 codes that passed FDR < 5% in the meta-analysis of either the highly consanguineous and/or full cohorts from G&H+UKB, as well as height. We first sought to calculate statistical power to replicate associations in the within-sibling analysis. Briefly, we simulated sibling pair F_ROH_ values and phenotypes with effect sizes equivalent to those detected in the highly consanguineous or full cohort analysis and derived empirical estimates of statistical power assuming the sample size available in 23andMe (STAR Methods). For the seven tested phenotypes that were significant in the highly consanguineous cohorts, we estimated we had 77% power to replicate at least one association and 38% power to replicate at least two associations at experiment-wide significance; for the seven that were only significant in the full cohorts, we had 52% power to replicate at least one association.

Height and the seven diseases that were significant in the G&H+UKB highly consanguineous cohorts showed convincing evidence of replication; across these eight phenotypes, we saw no significant difference between the effect sizes estimates in the 23andMe within-sibling analysis versus the analysis of highly consanguineous cohorts from G&H+UKB (mean ratio of β_within-sibling_/β_highly consanguineous_ = 0.913, p = 0.765 paired t test), there was no evidence for a bias of β_within-sibling_ < β_highly consanguineous_ (p = 0.688, exact binomial test), and all had concordant directions of effect size, significantly more than expected by chance (p = 0.004, one-sided binomial test). Additionally, two were experiment-wide significant: post-traumatic stress disorder (PTSD) (OR = 1.96 for F_ROH_ = 0.0625, 95% CI = [1.58, 2.43], p = 0.00082), which is included within ICD10 subchapter F43, and T2D (OR = 1.57 for F_ROH_ = 0.0625, 95% CI = [1.32, 1.86], p = 0.00395). We had 54% power to replicate height at p < 0.05, which did replicate at nominal significance (β = −0.99 cm, 95% CI = [−1.91, −0.074], p = 0.036). In contrast, of the seven tested diseases that were only significant in the G&H+UKB full cohorts, five had discordant directions of effect in 23andMe and none passed experiment-wide significance. Importantly, PTSD, the disorder with the most significant F_ROH_ association in the replication analysis, was only significant in the analysis of the highly consanguineous cohorts in G&H and UKB (Figure 4B).

### Population-attributable risk of autozygosity to T2D and asthma

British South Asians have more than twice the rate of T2D compared with White British Europeans,^13^^,^^33^ as well as a higher rate of asthma hospitalizations and death.^13^^,^^33^ Given the detected associations between autozygosity and these diseases, we estimated the fraction of the incidence of these disorders that may be attributable to autozygosity due to consanguinity in UKB EUR, British Pakistanis, and British Bangladeshis. To do so, we calculated the percent population-attributable risk (PAR) (i.e., percent of cases in the population attributable to autozygosity) for the two diseases (see STAR Methods). The calculation of PAR incorporates the prevalence of the risk factor, which we estimated from the fraction of individuals who were inferred to be offspring of first cousins or second cousins in the relevant cohort (Figure 1). It also incorporates the risk ratio for the disease, which we estimated based on the effect size estimated for F_ROH_ in the G&H+UKB meta-analysis of the highly consanguineous cohorts (Figure 4A; Table S3), and the prevalence of the disease in nonconsanguineous individuals. Since the latter is unknown, we varied the assumed prevalence from 5% to 15% for each disorder, as that should reasonably capture the true prevalence.^34^^,^^35^

**Figure 5:**
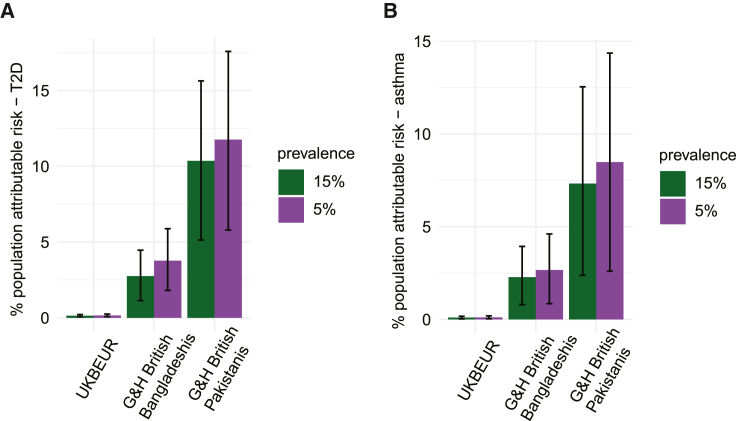
Population-attributable risk of F_ROH_ for T2D and asthma Percent population-attributable risk for (A) T2D and (B) asthma due to F_ROH_ estimated for UKB EUR and G&H British Bangladeshis and Pakistanis, assuming underlying prevalence estimates of disease in nonconsanguineous individuals equal to 5% or 15%. Error bars indicate 95% confidence intervals. See also Figure S4.

**Figure S4:**
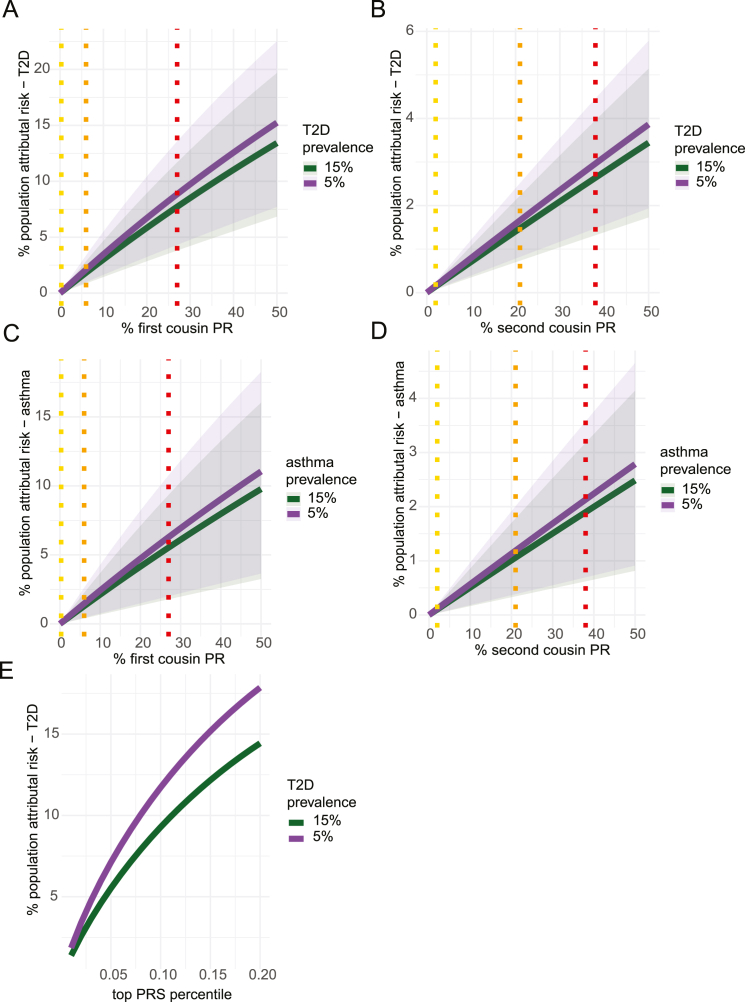
Percent population-attributable risk for T2D or asthma due to parental relatedness or T2D polygenic score, related to Figure 5 Percent population-attributable risk (PAR) for varying degrees of prevalence of parental relatedness for (A) and (B) T2D, and (C) and (D) asthma, and (E) for T2D for varying fractions of individuals in the top percentiles for T2D polygenic risk score (PRS). (A) and (C) show PAR owed to first cousin PR, and (B) and (D) for second cousin parental relatedness. Dotted lines indicate the population prevalence estimates for the indicated class of consanguinity in UKB EUR (yellow), G&H British Bangladeshis (orange), and G&H British Pakistanis (red). The prevalence of the disease among nonconsanguineous individuals was used to calculate the PAR for each disease using 5% and 15% prevalence, shown in purple and green, respectively. Shaded areas indicate 95% CI for the estimated PAR.

Assuming a 5% prevalence of disease in nonconsanguineous individuals, we estimated that 10.1% (5.2%–15.9%, 95% CI) of the prevalence of T2D in G&H British Pakistanis is attributable to autozygosity resulting from consanguinity (Figures 5 and S4A–S4D). This is independent of the environmental/cultural correlates of consanguinity that may influence risk of the disorder. The PAR was estimated at 2.6% (1.2%–4.6%) in G&H British Bangladeshis and at <1% in UKB EUR. Likewise, we estimated that 7.4% (2.5%–12.5%) of asthma cases in G&H British Pakistanis are attributable to autozygosity, 2.4% (0.9%–4.2%) in G&H British Bangladeshis, and <1% in UKB EUR. The estimates decrease slightly when assuming a prevalence of 15%. We conclude that a substantial proportion of the increased incidence of T2D in British Pakistanis is due to autozygosity resulting from consanguinity.

As a point of comparison for T2D, we considered the population-attributable risk due to having a high polygenic risk score (PRS) for the disease. We considered the T2D PRS developed by Mars et al.^36^ which showed similar predictive accuracy in cohorts of individuals with a majority European versus South Asian GIA (OR for 1 SD of the PRS ∼1.65 in both). In G&H British Pakistanis and British Bangladeshis, the increase in T2D prevalence due to autozygosity is similar to that due to individuals being in the top 5%–18% and 1%–3% of polygenic risk, respectively (Figure S4E).

### Impact of genetic architecture on F_ROH_ associations with binary traits

**Figure S5:**
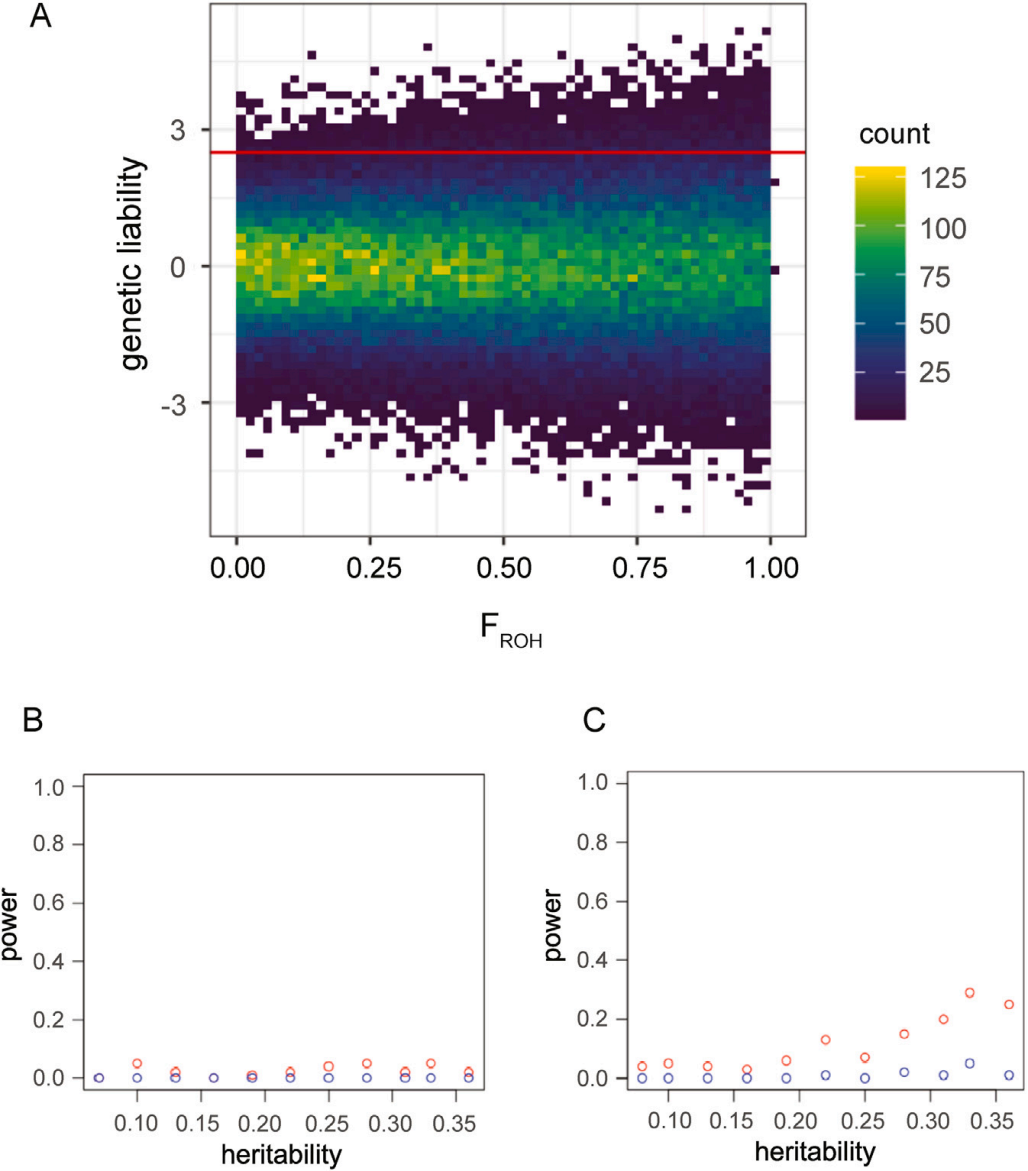
Associations between F_ROH_ and binary phenotypes under an additive architecture, related to Figure 4 and Table S3 (A) Demonstration of how a correlation between F_ROH_ and disease status can arise in a trait with solely additive genetic architecture. Here, we simulate additive genetic liability and F_ROH_ values for 100,000 individuals. The variance of additive genetic liability toward a trait increases with increasing F_ROH_. If we imagine that individuals with a genetic liability >2.5 (as shown by the red line) will be disease cases, more individuals will pass the threshold at higher values of F_ROH_ due to the increased variance in genetic liability. Thus, F_ROH_ could correlate with disease case status when the trait has a purely additive genetic architecture. (B and C) Power to detect significant associations between F_ROH_ and a binary trait with a purely additive genetic architecture and varying heritability. (B) with F_ROH_ values drawn from a lognormal distribution with variance of 0.5 and mean −2.5 and values restricted to be between 0.02 and 0.18 (i.e., mimicking the observed distribution in Figure 2B) and (C) shows the power to detect associations with F_ROH_ values drawn uniformly from 0 to 1. Red is the power for p < 0.05 and blue for p < 0.05/61. Power was determined with 100 simulations.

Associations between F_ROH_ and traits can be induced by several underlying genetic architectures. A commonly described hypothesis is that F_ROH_ increases the risk of inheriting deleterious recessive variants, thereby increasing genetic predisposition toward disease. An alternative (but not mutually exclusive) explanation is that autozygosity increases the additive genetic variance of a trait in the population (specifically by a factor of 1 + *F*, where *F* is the average “inbreeding coefficient” in the population,^17^ also see Note S1). Thus, under a liability threshold model for a binary trait, individuals with high values for F_ROH_ are more likely to cross the liability threshold even in the absence of non-additive effects, inducing an association between F_ROH_ and the trait (Figure S5A).

To assess the degree to which the increased additive variance could induce associations between F_ROH_ and diseases, we simulated binary traits with an additive polygenic genetic architecture and varying heritabilities, then estimated the power we would have to detect significant associations between F_ROH_ and the simulated traits in our current study, considering the sample size and F_ROH_ distribution in the highly consanguineous cohorts. We find that purely additive, polygenic traits with heritabilities similar to those of the most heritable traits we consider (e.g., T2D with an estimated narrow-sense h^2^ of 20%–30%^37^) would be very underpowered to show significant associations with F_ROH_ in our study (Figure S5B). In the unrealistic case of F_ROH_ values in the population being uniformly distributed from 0 to 1, there would still be very little power to detect associations at our current sample size (Figure S5C). We conclude that the associations we observe are unlikely to reflect a solely additive genetic architecture. Hence, our findings highlight the possibility of widespread non-additive effects on diseases across the phenotypic spectrum.

## Discussion

We introduce a robust approach to reduce confounding in studies assessing trait associations with autozygosity by restricting analyses to highly consanguineous individuals. We find compelling evidence that autozygosity impacts several common diseases spanning multiple organ systems, notably T2D and PTSD. Simulations indicate that the associations most likely stem from non-additive genetic effects, and we calculate population-attributable risk fractions to show that these effects cumulatively contribute substantially to disease incidence in communities with high rates of consanguinity.

In concordance with previous studies,^1^^,^^38^^,^^39^ we find that British Bangladeshi and Pakistani individuals practice consanguinity at higher rates than British individuals with majority European GIA. Our results from G&H show that younger British Pakistanis are more likely to have parents who are inferred to be first cousins. One might be concerned that this observation could be due to the impact of autozygosity on health, which could lead to an ascertainment bias whereby younger people would be more likely to be recruited to the study if they had higher autozygosity and were hence less healthy and spent more time in healthcare settings where much of the recruitment took place. However, the fact that we see the opposite pattern (i.e., a positive association between age and likelihood of consanguinity) in British Bangladeshis suggests that ascertainment bias is not driving the association between consanguinity and age that we see in British Pakistanis. We cannot be sure whether the patterns we observe are due to changing patterns of unions within the UK across time or temporal changes in migration rates from Pakistan/Bangladesh to the UK that affected trans-national marriage/union patterns.^40^ Recent work in large biobank settings has shown that overall rates of consanguinity are decreasing in large cohorts from the United States (All of Us and the Million Veterans Program) and increasing in UKB South Asians.^28^ Our analysis suggests that examining these trends at the level of a whole country or broad-scale genetic ancestry group (e.g., South Asian) may obscure fine-scale differences. Also, considering only changes in mean F_ROH_ may obscure changes in rates of different types of consanguinity (Figures 1 and S1). These results highlight important trends for clinical settings, as autozygosity increases the risk of recessive Mendelian diseases^41^ and, as we show here, several common, complex disorders.

Before we assessed associations between F_ROH_ and disease, we investigated associations between F_ROH_ and common confounders that are associated with disease risk, including socioeconomic, behavioral, and cultural traits in UKB. When considering all individuals, we found significant associations between F_ROH_ and university attendance, deprivation, religiosity, and alcohol/tobacco use (Figure 3). All of the associations were attenuated by our approach of restricting analysis to the highly consanguineous cohort, suggesting that they were due at least in part to confounding. Consistent with this, religiosity and tobacco use were likewise not significant in the 23andMe within-sibling analysis. We also considered whether, in aggregate, these confounders plus exercise- and diet-related variables explain a significant amount of variance in F_ROH_ within UKB EUR and UKB SAS; we found a significant portion of variance is explained in the full cohorts but not in the highly consanguineous cohorts (Table S1). We note that we may have reduced power to detect confounding associations in the highly consanguineous cohorts, especially those of potentially weaker effect; this led us to conduct further robustness checks of the disease associations, namely redoing the analysis in UKB controlling for measured confounders and the PR-stratified meta-analysis (Table S4), both of which suggested that our results were robust. We found that conditioning on EA, a sensitivity analysis common in the autozygosity literature,^9^^,^^10^^,^^30^ did not attenuate the associations between F_ROH_ and the potential confounders assessed (Figure 3). These analyses illustrate the need to carefully assess whether the causes of F_ROH_ associations in several previous studies are indeed biological and emphasize that they should be interpreted with caution.

Having demonstrated that restricting analyses to highly consanguineous individuals greatly attenuates confounding, we investigated associations between F_ROH_ and clinical phenotypes extracted from EHRs within this group. We found significant associations between F_ROH_ and twelve diseases classified by three-digit ICD10 codes, including T2D, asthma, and two psychiatric disorders (“F43—reaction to severe stress disorders,” which includes PTSD, and “F41—other anxiety disorders”). These included several that are not well-defined diseases, such as shoulder lesions, which include adhesive capsulitis, a common comorbidity of T2D.^42^ It has also been shown that PTSD symptoms and diagnosis are associated with increased risk for T2D.^43^ As cohorts with highly consanguineous individuals grow and non-additive loci are discovered for these disorders, it may be possible to disentangle the potential causal paths operating between these associations.

There are multiple risk factors for the diseases for which we found significant F_ROH_ associations that differ between British South Asians and White British people (e.g., diet and lifestyle factors for T2D,^44^^,^^45^^,^^46^^,^^47^^,^^48^^,^^49^ socioeconomic status and experience of racial discrimination for PTSD^50^^,^^51^). However, there are several reasons we feel these are unlikely to confound our results here. Firstly, for the measured confounders we were able to assess in UKB, we saw no evidence of significant associations with F_ROH_ within the highly consanguineous group (Table S1). Secondly, we replicated the associations between F_ROH_ and both T2D and PTSD at experiment-wide significance in the 23andMe within-sibling analysis, which is free from confounding because variation in F_ROH_ within families is randomly determined at conception and is not expected to be correlated with environmental confounders. We showed that limited power was the likely reason that some of the findings from the highly consanguineous group did not replicate in 23andMe.

When analyzing the full cohort from G&H+UKB, we found multiple additional associations. However, when attempting to replicate seven of these via within-sibling analysis in 23andMe, none passed experiment-wide significance, and five had discordant directions of effect size, indicating that they were likely spurious. Interestingly, the analysis of the full G&H+UKB cohorts gave nonsignificant results for the two psychiatric disorders identified in the highly consanguineous analysis (Figure 4B). This result suggests that environmental/cultural factors correlated with consanguinity, and therefore, F_ROH_, in these cohorts, are either truly protective against these disorders and/or that consanguineous individuals are less likely to seek medical assistance for them. Thus, our approach not only addresses spurious associations between F_ROH_ and diseases but also prevents masking that is potentially due to consanguinity-related differences in disease ascertainment in EHRs.

We showed that the risk of T2D and asthma incurred by autozygosity may contribute substantially to the incidence of these diseases in British Pakistanis and, to a lesser degree, in British Bangladeshis. Our estimates of PAR assume that G&H is representative of the broader British Pakistani and Bangladeshi communities in the UK, although we note that the majority of the current G&H cohort is from London. Our recent work in a cohort collected in Bradford, a city in the north of England with a substantial British Pakistani population, reported higher rates of consanguinity than found here, with 44% of British Pakistanis inferred to have parents who are first cousins or closer^2^ (compared with 33% in the current study), suggesting we are potentially underestimating the true PAR. For T2D, we found that the rate of consanguinity in British Pakistanis increases the prevalence approximately equivalently to individuals being in about the top decile of common variant risk measured in a previous study (Figure S4E).^36^ Importantly, we note that our estimates for the PAR due to autozygosity have large standard errors (the CIs for T2D for British Pakistanis span between 5.2% and 17.5%, depending on assumed prevalence) and that other risk factors for T2D have a far higher PAR than autozygosity. One study estimated the PAR for having BMI > 25 kg/m^2^ is >60% in the Americas, with little fluctuation between geographic regions.^52^ In a separate study of a cohort based in Rotterdam, the PAR for BMI > 25 kg/m^2^ was 51%, and 71% for all modifiable risk factors assessed in their study (high BMI and waist circumference, current smoking, and high C-reactive protein).^53^ Thus, although the impact of autozygosity resulting from consanguinity on T2D risk is significant, its impact is less substantial than that of other risk factors that are modifiable. Furthermore, the health risks incurred by consanguinity need to be weighed against potential social and economic benefits for communities.

Via simulations, we show that the associations we detected are unlikely to be due to autozygosity increasing additive variance for genetic risk of binary traits, suggesting widespread non-additive effects. In the few studies that have looked, recessive-acting rare and common variants have been found to be associated with multiple common diseases including T2D.^54^^,^^55^^,^^56^ However, it has been previously shown that dominance heritability at common variants is negligible,^57^^,^^58^ suggesting that the observed F_ROH_ associations likely stem from non-additive effects at low allele frequency variants and/or epistasis. It has been suggested that comparing the effect sizes of F_GRM_ and F_ROH_ distinguishes whether associations are driven by homozygosity at common versus rare variants.^9^ We found that the F_GRM_ effect size estimates in the highly consanguineous cohorts were concordant with those of F_ROH_, although with a weaker effect, with the ratio of effect sizes F_GRM_/F_ROH_ being significantly less than 1 (0.915, p = 2 × 10^−6^, paired t test) (Table S4). This could be interpreted as evidence that the F_ROH_ associations are more likely driven by rarer variants. However, one alternative explanation for this could be that F_GRM_ is more prone to misestimation than F_ROH_, as F_GRM_ depends on estimates of allele frequencies in the population and will be biased for individuals who are more poorly represented in the reference group used to estimate allele frequencies. Having said this, we note the correlations between F_GRM_ and F_ROH_ are ∼0.95 in the highly consanguineous cohorts.

Our study indicates that non-additive effects contribute to disease risk, but mapping specific loci will require large cohorts. Assuming an outbred population, detecting recessive effects requires much larger sample sizes than for additive loci, since only *np*^2^ individuals have informative alternative genotypes (where *n* is the sample size and *p* is the effect allele frequency) versus *n*(*p*(1 − *p*) + *p*^2^)) = *np* under an additive model. This issue is especially exacerbated at rare variants due to the quadratic scaling but is reduced in consanguineous cohorts where the number of informative alternative genotypes for recessive loci is *n*((1 − *F*)*p*^2^ + *Fp*) (where F is the average F_ROH_ in the sample). Thus, large sequenced cohorts enriched for consanguineous individuals will be necessary to fully characterize the nature of non-additive genetic effects across the allele frequency spectrum on polygenic traits.

In conclusion, we have described patterns of consanguinity in two large UK cohorts and proposed a robust approach to control for social and environmental confounding in autozygosity association studies. We found multiple significant associations between autozygosity and common diseases that we contend are unlikely to be confounded. Our findings suggest that previous results in the field should be revisited, as they may have been driven by uncontrolled confounders. Furthermore, our results indicate that autozygosity may be an important contributing factor to the increased incidence of T2D in British Pakistanis as well as in other worldwide populations with high rates of consanguinity. Our work motivates the incorporation of genome-wide autozygosity into predictions of genetic risk as well as a search for individual non-additive-acting variants and genes influencing disease risk across the phenotypic spectrum.

### Limitations of the study

Our paper has several limitations. Our approach assumes that within the highly consanguineous subset of the cohort, the degree of autozygosity is not correlated with environmental factors that influence disease risk, which we cannot totally rule out. Our results suggest that there are no significant associations with some obvious potential measured confounders within this group, but these confounders may still be associated with F_ROH_ with weaker effect sizes than we are powered to detect, or there may be other confounders that were not measured. Another limitation is that we were underpowered to replicate many of our findings in the within-sibling analysis from 23andMe; having said that, we did replicate more than we would have expected to at 80% power. Results for the diseases we did not replicate should be treated with caution unless replicated in future studies. Furthermore, our results may not necessarily generalize outside the set of highly consanguineous individuals we have studied. For example, we noted non-linear effects of F_ROH_ for individuals with extremely high values (whom we had excluded from our main analysis) (Figure S2B), which could indicate extreme environmental confounding^59^ but might also be partly due to the increased potential for epistasis between rare homozygous variants.^60^ Additionally, we have not assessed sex-stratified F_ROH_-phenotype associations, which would be an interesting avenue for future work.

## Consortia

The current members of Genes & Health Research Team (in alphabetical order by surname): Shaheen Akhtar, Mohammad Anwar, Elena Arciero, Omar Asgar, Samina Ashraf, Gerome Breen, Raymond Chung, Charles J. Curtis, Shabana Chaudhary, Maharun Chowdhury, Grainne Colligan, Panos Deloukas, Ceri Durham, Faiza Durrani, Fabiola Eto, Sarah Finer, Ana Angel Garcia, Chris Griffiths, Joanne Harvey, Teng Heng, Qin Qin Huang, Matt Hurles, Karen A. Hunt, Shapna Hussain, Kamrul Islam, Ben Jacobs, Ahsan Khan, Amara Khan, Cath Lavery, Sang Hyuck Lee, Robin Lerner, Daniel MacArthur, Daniel Malawsky, Hilary Martin, Dan Mason, Mohammed Bodrul Mazid, John McDermott, Sanam McSweeney, Shefa Miah, Sabrina Munir, Bill Newman, Elizabeth Owor, Asma Qureshi, Samiha Rahman, Nishat Safa, John Solly, Farah Tahmasebi, Richard C. Trembath, Karen Tricker, Nasir Uddin, David A. van Heel, Caroline Winckley, and John Wright.

The following members of the 23andMe Research Team contributed to this study: Stella Aslibekyan, Adam Auton, Elizabeth Babalola, Robert K. Bell, Jessica Bielenberg, Katarzyna Bryc, Emily Bullis, Daniella Coker, Gabriel Cuellar Partida, Devika Dhamija, Sayantan Das, Sarah L. Elson, Nicholas Eriksson, Teresa Filshtein, Alison Fitch, Kipper Fletez-Brant, Pierre Fontanillas, Will Freyman, Julie M. Granka, Karl Heilbron, Alejandro Hernandez, Barry Hicks, David A. Hinds, Ethan M. Jewett, Yunxuan Jiang, Katelyn Kukar, Alan Kwong, Keng-Han Lin, Bianca A. Llamas, Maya Lowe, Jey C. McCreight, Matthew H. McIntyre, Steven J. Micheletti, Meghan E. Moreno, Priyanka Nandakumar, Dominique T. Nguyen, Elizabeth S. Noblin, Jared O'Connell, Aaron A. Petrakovitz, G. David Poznik, Alexandra Reynoso, Morgan Schumacher, Anjali J. Shastri, Janie F. Shelton, Jingchunzi Shi, Suyash Shringarpure, Qiaojuan Jane Su, Susana A. Tat, Christophe Toukam Tchakouté, Vinh Tran, Joyce Y. Tung, Xin Wang, Wei Wang, Catherine H. Weldon, Peter Wilton, and Corinna D. Wong.

## STAR★Methods

### Key resources table

**Table:** 

REAGENT or RESOURCE	SOURCE	IDENTIFIER
**Deposited data**		

1000 Genomes Project whole genome sequence data	Fairley et al.^61^	https://www.internationalgenome.org/data
Genes & Health genotype array data	Finer et al.^27^	https://www.genesandhealth.org/research/scientists-using-genes-health-scientific-research
UKBiobank genotype array data	Bycroft et al.^62^	https://www.ukbiobank.ac.uk/enable-your-research/apply-for-access

**Software and algorithms**		

bife	Stammann et al.^63^	https://cran.r-project.org/web/packages/bife/index.html
consanguinity_simulation	Arciero et al.^2^	https://github.com/malawsky/consanguinity_simulation
KING	Manichaikul et al.^64^	https://www.kingrelatedness.com/
PLINK 1.9	PLINK Working Group	https://www.cog-genomics.org/plink/1.9/
plm	Croissant and Millo^65^	https://cran.r-project.org/web/packages/plm/index.html
R 4.0.2	R Core Team	https://www.r-project.org/

### Resource availability

#### Lead contact

Further materials and requests may be directed to lead contact, Hilary Martin (hcm@sanger.ac.uk).

#### Materials availability

This study did not generate new unique reagents.

### Experimental model and study participant details

#### G&H cohort

The G&H cohort was recruited across several sites in East London, Luton, Manchester, and Bradford, including community settings (e.g. mosques, shopping centres, libraries) and primary care clinics.^27^ Fifty six percent of individuals were recruited in primary care settings, 5% were recruited in hospitals, and the remainder were recruited in community settings. Information regarding sample size, fraction of male/female participants, self-reported ethnic background, and age distribution can be found in Table 1.

#### UK Biobank cohort

The UK Biobank is a prospective cohort of over 500,000 individuals with genotype and deep phenotype data recruited across the United Kingdom between 2006 and 2010.^62^ Information regarding sample size, fraction of male/female participants, self-reported ethnic background, and age distribution can be found in Table 1.

#### Saudi Arabian cohort

The Saudi Arabian cohort is a cohort of 5,668 Saudi Arabian individuals recruited at the Institutional Catheterization Centre in the King Faisal Specialist Hospital and Research Centre.^66^ The cohort is 64% male with mean age 54.8 years (standard deviation 14.8 years). Individuals have genotype and phenotype data collected from medical records and interviews conducted by trained healthcare professionals at the hospital.

#### 23andMe cohort

The 23andMe cohort is a prospective cohort of over 8 million research consented individuals, with genotype array and self-reported phenotype data. In the current study, we used research-consented individuals with at least one genetically-inferred full sibling also in the cohort (see STAR Methods: section within-sibling analysis in 23andMe). The total sample size of 23andMe individuals analysed in the current study is 545,806.

#### Ascertainment of sex across cohorts

Participants in the G&H, UK Biobank and Saudi cohorts were asked their sex with at least the options of ‘female’ or ‘male’ (some cohorts include other sex options) and had their sex genetically inferred via genotype microarray data (male having XY and female having XX chromosomes). Individuals with discordant self-reported and genetically-inferred sex were removed to prevent the chance of sample mixups. In 23andMe, sex was similarly genetically inferred via genotype microarray data.

### Method details

#### G&H, UK Biobank, and Saudi Arabian cohorts genotype data preparation

We used the 2021 July data release of the G&H data, which contained 46,132 individuals genotyped on the Illumina Global Screening Array v3EAMD (GRCh38). We first removed 1,736 individuals with call rate less than 99.2% and SNPs with MAF < 1%, leaving 355,862 SNPs. To ensure we did not lose SNPs that have high quality but that fail Hardy-Weinberg Equilibrium due to high rates of consanguinity and strong population structure in British Pakistani individuals, we removed 726 SNPs that failed Hardy-Weinberg Equilibrium p-value < 1x10^-6^ in British Bangladeshi individuals alone, as done in Huang et al.^67^ This left 355,136 SNPs.

Genotyping and processing for the UK Biobank cohort were done centrally by the UKB group.^62^ Two customized Affymetrix genotyping arrays were used, the UK Biobank Axiom array (n=438,692) and the UK BiLEVE Axiom array (n=50,520), which covered 812,428 SNPs with 95% overlap between the arrays. Quality control consisted of excluding individuals with >3% missingness, inconsistent sex, sex aneuploidy, excess heterogeneity, or withdrawn consent.

For the Saudi Arabian cohort, genotyping and processing has been described previously.^66^ Briefly, individuals were genotyped on the Affymetrix Genome-Wide ASI Array including 598,000 SNPs. SNPs with call rate <95% and HWE p-value <1x10-6 and individuals with >5% missingness were excluded, resulting in 537,798 SNPs.

In the three cohorts, we estimated the relatedness between individuals using PropIBD from KING^64^ removed one from each pair of related individuals inferred to be 3^rd^ relatives or closer. To remove related individuals while maximising the sample size, we ranked individuals by their number of relatives, then removed the individual with the highest number of relatives iteratively until no relatives remained.

### Quantification and statistical analysis

#### Inference of genetic ancestry

In G&H, we determined genetically-inferred ancestry (GIA) by merging the data with reference sequences of unrelated individuals (determined using KING as described above) from the 1000 Genome Project^68^ and Central and South Asian individuals from the Human Genome Diversity Project.^69^ We first excluded palindromic variants and multiallelic sites from both datasets. Then, we merged the external reference data and G&H by matching positions and alleles of the common SNPs that passed QC in G&H, and kept variants found in both datasets, which left 349,632 SNPs. A further 1285 variants were excluded due to AF discrepancies between G&H and South Asian reference individuals (>4 standard deviations from the mean residual of -log_10_ frequency bins, and Fisher's exact test p<1x10^-5^), resulting in 348,347 variants. PLINK 1.9 LD pruning was performed with a window size of 1000kb, step size 50 and LD r^2^ cutoff of 0.1, then long LD regions^70^ were excluded, resulting in 104,552 variants. We used PLINK 1.9 to calculate principal components (PCs). We first calculated PCs for the 3,433 reference individuals, then projected the G&H individuals into the reference PC space. We calculated UMAP coordinates using the umap R package. We found that the UMAP with 7 PCs was optimal to separate the reference individuals into groups that corresponded to the continents from which their recent ancestors originated. 44,320 out of 44,396 G&H individuals were most genetically similar to the reference South Asian individuals, as measured by their proximity on this UMAP plot, and were classed as having South Asian GIA. Amongst these, we then performed a second PC analysis on the unrelated G&H individuals, projecting the related G&H individuals into the PC space defined by the unrelateds. A UMAP with 4 PCs identified two large clusters which largely corresponded to individuals who self-reported as being of Pakistani or Bangladeshi origin respectively, plus a couple of smaller clusters which we excluded henceforth. This was used to classify individuals in the GIA groups which we refer to as “British Pakistani” and “British Bangladeshi” throughout (total N=44,190 in the final dataset).

UKB individuals were projected into the 1000 Genomes PCA space, which defined five GIA groups that corresponded to the continents from which the 1000 Genomes individuals’ recent ancestors came. UKB individuals were then assigned to the GIA group to which they had the highest genetic similarity, based on the Mahalanobis distance between their position in PC space using 6 PCs and the average position of each 1000 Genomes GIA group. Individuals with a Mahalanobis distance that deviated from each GIA group average by >6 standard deviations were excluded. 387,531 individuals of majority European GIA (UKB EUR) and 9,653 individuals of majority South Asian GIA (UKB SAS) remained after quality control.

#### ROH calling

For ROH calling in G&H, we filtered out SNPs with minor allele frequency <5% and used PLINK 1.9 to call ROHs on the filtered SNPs using the following parameters, following Clark et al.^9^: --homozyg-window-snp 50 --homozyg-snp 50 --homozyg-kb 1500 --homozyg-gap 1000, --homozyg-density 50 --homozyg-window-missing 5 --homozyg-window-het 1. In UKB we followed the same procedure, but before ROH calling we removed variants that had Hardy-Weinberg p<1x10^-6^ in the relevant GIA group (UKB EUR or UKB SAS).

We calculated F_ROH_ by summing up the total length of all autosomal ROHs previously calculated (in base pairs) and dividing by 2.7 billion (the approximate length of the autosomal genome), following Clark et al.^9^

#### Consanguinity inference

We used the method to infer parental relatedness which we described previously.^2^ Briefly, unrelated individuals were randomly chosen from the actual dataset, phased using EAGLE, v2.4.1,^68^ and pedigrees are simulated using custom R code available at https://github.com/malawsky/consanguinity_simulation; specifically we included the following PR categories: unions between individuals who are siblings, avuncular pairs (including multiple generations), first cousins (including multiple generations), first cousins once removed, and second cousins, as well as between unrelated individuals. We then applied the same ROH calling procedures described above to the simulated offspring. For each simulated individual, we then calculated fifteen statistics for the purposes of classification using a neural net classifier: the total length of the ten longest ROHs (in cM), and the frequency of ROHs ranging from 10 to 150 cM binned into 14 intervals of 10 cM. Using these statistics, we trained a neural net classifier implemented in the R package *nnet* to assign simulated individuals to a given PR category by repeating this procedure 10 times, summing up the probabilities for each possible PR category, and choosing the one with the highest probability per individual. We then calculated the same statistics on the true samples and used the trained neural net classifier to infer the degree of PR. For most of our analyses, we group together people whose parents were inferred to be second cousins with first cousins once removed, and people whose parents were inferred to be first cousins for one/two/three generations, because of the low accuracy in differentiating between the finer-grained classifications.

#### Analysis of consanguinity patterns in G&H and UK Biobank

In G&H, individuals were asked about their parental relatedness at recruitment (“Were your parents related by blood? (not just by marriage)”) with the options of “Yes”, “No”, and “Don't know”. If the individual answered “Yes”, they were asked a follow-up question of “If Yes, how were your parents related?” with the options of “First Cousins”, “Don't Know'', and “Other related by blood”. Figures 1B and 1C shows the inferred degree of parental relatedness for individuals split by self-reported parental relatedness.

We used linear regression to regress F_ROH_ on age G&H British Pakistanis, G&H British Bangladeshis, UKB EUR and UKB SAS, controlling for sex and 20 PCs. To test if overall consanguinity changed over time, we made a binary variable indicating parental relatedness (1 if inferred to have parents that are second cousins or closer, 0 otherwise) and regressed that on age, sex, and 20 PCs using a logistic regression. To test for more subtle changes in consanguinity patterns over time, we made a categorical variable indicating each of the three main inferred parental relatedness categories (first cousins or closer, second cousins/first cousins once removed, or unrelated), and regressed it on age, sex, and 20 PCs using a multinomial logistic regression with the nnet R package.^71^

#### Phenotypic data harmonisation and preparation for G&H

The G&H EHR data consisted of SNOMED codes from primary care data for 34,712 of the participants (i.e. those registered with a GP in inner London, outer London, and Bradford), ICD10 codes from secondary care data for 17,132 individuals (i.e. those who had attended the Barts Health or Bradford University Hospitals NHS trusts), and ICD10 codes from national Hospital Episode Statistics available on all participants. There were twelve participants with no ICD10 codes, and we removed these individuals from the analyses since it was possible that they had recently moved to the UK so may be missing any EHR data for that reason. After removal of relatives, 23,978 individuals were retained. We translated SNOMED codes in primary care data to ICD10 codes using the Interactive Map-Assisted Generation of ICD10 Codes algorithm (using only codes with strict 1:1 mapping, as also done by UK Biobank).^72^

Our methods were designed to closely resemble those used in UK Biobank. For each ICD10 code, we determined whether the participant had any diagnostic codes equivalent to the ICD10 code, the date of the earliest diagnostic code, and the data sources which corroborated the presence of the ICD10 code. In total, we combined data from the following different sources: Barts Health inpatient and outpatient care (native format ICD10, n=23,940 unique pseudoNHS numbers with >=1 code, clinical coding), Barts Health inpatient and outpatient care (native format SNOMED description IDs, n=20,967 unique pseudoNHS numbers with >=1 code, directly coded by healthcare professionals), Bradford Teaching Hospitals inpatient and outpatient care (native format ICD10, n=1,615 unique pseudoNHS numbers with >=1 code, clinical coding), Bradford Teaching Hospitals inpatient and outpatient care (native format SNOMED description IDs, n=1,740 unique pseudoNHS numbers with >=1 code, directly coded by healthcare professionals), primary care observations from the Discovery Clinical Commissioning Group (CCG) and Tower Hamlets (native format SNOMED concept IDs, n=39,077 unique pseudoNHS numbers with >=1 code, coded directly by primary care professionals), NHS Digital Hospital Episode Statistics (both Admitted Patient Care and Outpatient Care), and mortality records (native formats ICD10).

First, we mapped SNOMED description IDs to SNOMED concept IDs for clinician-coded SNOMED codes pertaining to participants who had healthcare encounters at Bradford Teaching Hospitals or Barts Health. The SNOMED mapping file was downloaded from the NHS Digital website on 12/05/22. We used SNOMED build SNOMEDCT2_32.12.0_20220413000001 – the 20th April 2022 minor release (fileset uk_sct2cl_32.12.0_20220413000001Z.zip). This folder contains four separate link files referring to the international SNOMED edition and three distinct UK-specific editions. These files contain mapping for SNOMED descriptionIDs to SNOMED conceptIDs. We collated them into a single mapping reference. All description IDs map onto a single conceptID. This relationship is many-to-one: each descriptionID maps to a single conceptID, but each conceptID can be referred to by several descriptionIDs (the median is three). In total we used a mapping reference consisting of 1,746,657 unique SNOMED description IDs mapping to 578,387 unique SNOMED concept IDs.

For the Barts Health data, we obtained three separate datasets containing records of ‘Diagnoses’, ‘Problems’, and ‘Procedures’ respectively. These files were merged with the mapping files based on the description ID. We excluded codes with a missing SNOMED description ID. Overall we were able to successfully map a high proportion of SNOMED description IDs to concept IDs:

- Diagnoses: 118191 out of 138235 records mapped (85.5%)
- Problems: 31006 out of 31084 records mapped (99.75%)
- Procedures: 3518 out of 3586 records mapped (98.1%)

The most common unmapped code was a code for ‘Venous Thromboembolism Risk Assessment’ (n=13,887 codes), an administrative code of no diagnostic relevance, referring to a standard thromboembolism risk checklist completed on patient admission within Barts Health. Exclusion of this code improved the mapping for the diagnoses dataset from 85.5% to 95.2%. We performed identical mapping for Bradford Teaching Hospitals ‘Diagnoses’ and ‘Problems’ data with a similar successful mapping percentage.

Next, we mapped these codes to ICD10 using the most recent SNOMED maps from NHS digital (SnomedCT_InternationalRF2_PRODUCTION_20210131T120000Z and SnomedCT_UKClinicalRF2_PRODUCTION_20220413T000001Z). We combined the UK and the international map. We restricted this map to SNOMED concept IDs which mapped to a single 3-digit ICD10 code (i.e. a 1-to-1 relationship), resulting in 119,459 individual SNOMED concept IDs. We combined the derived SNOMED concept IDs from step 1 with ‘directly coded’ ICD10 data for each participant in Barts Health and Bradford data separately. 4-digit ICD10 codes were truncated to the first three characters. We then processed data from two primary care networks: the Discovery Clinical Commissioning Group (CCG) network and Tower Hamlets. These data were provided as SNOMED concept IDs and were mapped to ICD10 codes using the same 1:1 mapping approach as for primary care data. Overall between 3% and 8% of all primary care codes were successfully mapped to ICD10 codes, reflecting the large number of administrative and measurement codes recorded in primary care, e.g. ‘text message sent to patient’, ‘blood pressure recording’, and ‘body mass index’.

We then combined 3-digit ICD10 codes derived from these sources (primary care, Barts Health, Bradford Teaching Hospitals) with data exports from NHS Digital (mortality records, HES outpatients and HES APC). Mortality records were searched for underlying cause of death (provided in ICD10 3-digit format). HES-APC codes were used to extract all diagnostic codes recorded during an admission (provided in ICD10 format). We used the admission date as the date of the report. HES-OP data were used to extract all diagnostic codes recorded in relation to the appointment, also provided in ICD10 format. The appointment date was used as the date of report. All ICD10 codes were truncated to 3-digit codes. We excluded ICD10 codes describing generic symptoms rather than disease entities (codes beginning R-Z). For each ICD10 code and each participant, we determined the presence/absence of the ICD10 code (in any health records), the data sources supporting the presence of the code, and the earliest recorded code. When determining the earliest reported code we excluded codes which encode ‘special dates’ in electronic healthcare records (placeholders for missing data) - 1/1/1860, 30/12/1899, 31/12/1899, and 1/1/1900. Similarly to UKB, we derived the ‘source of first report’ field by taking the earliest reported source for the ICD code and specifying whether other data sources supported the code. e.g. if an individual has a diagnostic code for G35 in primary care records and Barts Health data, with the first primary care code being recorded earlier, their ‘source of first report of G35’ value would be ‘Primary care and other sources’. For simplicity, we grouped data sources into ‘secondary care’, ‘primary care’, and ‘mortality’.

Overall, we successfully mapped data for 46,279 unique NHS numbers, 1,926 unique 3-digit ICD10 codes, and 2,976,436 individual diagnoses.

Since we suspected that coding practices might be different in different areas, and since missing EHR data could otherwise affect our results, in the analyses described below we included indicator variables to account for:

- Whether a G&H individual had primary care data from an inner London borough, outer London borough, and/or Bradford (3 binary variables)
- Whether a G&H individual had at least one secondary care code from Barts Health or Bradford University Hospitals NHS trust (2 binary variables)

#### Phenotype preparation for UK Biobank

To define disease phenotypes in UKB (Figure 4; Tables S3 and S4), we used the ‘first-occurence’ ICD10 codes (field 1712). The UKB phenotypes used in Figure 3 and Tables S1 and S2 were as follows:

• Religiosity (field 100328) indicates whether an individual reported attending a religious group at least once a week.
• Townsend deprivation index (field 189) was used as a proxy for socioeconomic status.
• Educational attainment (field 6138) was binarised into ‘having attended university’ or not when used as an outcome phenotype (for easy of comparison with the other phenotypes in Figure 3), but when used as a covariate (right hand side of Figure 3), we converted it to ‘years in education’ as done previously.^73^
• ‘Ever drinking alcohol’ was obtained from field 1558.
• ‘Ever smoked’ was obtained from field 20160.
• ‘Exercise’, quantified as the number of days/week of moderate physical activity for 10+ minutes, was obtained from field 884.
• BMI and height were obtained from fields 21001 and 50, respectively.
• Dietary PCs were calculated as done in Cole et al.^74^ separately in UKB EUR and UKB SAS. We conducted PCA on food preferences from the food frequency questionnaire using fields 20117, 1618, 6144, 1418, 1428, 1448, 1508, 1289, 1299, 1309, 1319, 1438, 1458, 1488, 1498, 1528, 1578, 1329, 1339, 1349, 1359, 1369, 1379, 1389, 1408, 1478, 1518, 1558, 1618, and 1418. Categorical questions were dummy coded, resulting in 85 variables. We used prcomp in R to run PCA on those variables and extract the first 5 PCs.

#### Regression analyses in G&H and UK Biobank

We considered two subsets of individuals in each cohort (G&H, UKB and UKB SAS) to identify associations between F_ROH_ and phenotypes: the full cohort, including all individuals, and the highly consanguineous cohort, consisting of individuals inferred to be offspring of first cousin or avuncular unions. (In practice, the vast majority of these were inferred to be offspring of first cousins). We used logistic regression in base R for binary variables.

For G&H, as covariates in the regression we included F_ROH_, sex, age, age^2^, age^∗^sex, genetic PCs 1–20, (has primary care code from outer London primary care data), (has primary care code from inner London primary care data), (has primary care code from Bradford), (has secondary care code from Barts Health), and (has secondary care code from Bradford University Hospitals NHS Trust).

In UKB, slightly different covariates were used, including F_ROH_, sex, age, age^2^, age^∗^sex, genetic PCs 1-20, array (UKB field 22000), batch (UKB field 22000), recruitment centre (UKB field 54), and whether primary care data were available (UKB field 42040).

For the meta-analysis of disease phenotypes, we used inverse variance-weighted fixed effect meta-analysis of estimates obtained from regressions in the UKB EUR and UKB SAS cohorts and in G&H.

For the log(OR) estimates by residualised F_ROH_ quintiles (Figure S2), we regressed F_ROH_ on the other covariates and binned F_ROH_ values by quintiles. The quintiles were defined in the G&H highly consanguineous cohort, as the F_ROH_ distribution in this group was very similar to that seen in the highly consanguineous individuals from UKB. We then regressed a given trait on the binned residualised F_ROH_ quintiles and meta-analysed the effect size across the three cohorts. For a linear regression of the log(OR), we used an inverse variance-weighted linear regression using SE estimates for each log(OR) estimate. We followed the same procedure in the full cohort (Figure S2B), but instead used the following bins for residualised F_ROH_: [0, 0.0001), [0.001, 0.002), [0.002, 0.003), [0.003, 0.004), [0.004, 0.005), [0.005, 0.006), [0.006, 0.007), [0.01, 0.02), [0.02, 0.03), [0.03, 0.18), [0.18, 1). The [0.03, 0.18) bin contains >95% of individuals in the highly consanguineous cohorts.

We also conducted a sensitivity analysis in which we stratified each highly consanguineous cohort into three groups: individuals inferred to have 1, 2, or 3 generations of first cousin parental relatedness. We used the same covariates as before, and, within each cohort (UKB EUR, UKB SAS and G&H), conducted a variance-weighted fixed effect meta-analysis to derive a PR-stratified effect size estimate for each cohort. We then meta-analysed these values across the three cohorts.

#### Analyses to assess power to detect associations with confounders in UKB

We used G^∗^Power^29^ to calculate power to detect a significant effect size of F_ROH_ on a given putative confounder in a logistic regression in the highly consanguineous cohorts, assuming the effect size estimates observed in the full cohorts (Figure 3). For each trait, we used the OR estimated in the full cohort analyses, the frequency of the binary phenotype in the highly consanguineous cohort, the sample size for a given highly consanguineous cohort (UKB EUR or UKB SAS), and a p-value threshold of 0.05. We assumed that F_ROH_ followed a log-normal distribution for with mean -2.5 and standard deviation of 0.5, with F_ROH_ values restricted to be between 0.02-0.18 (which approximates the empirical distribution of F_ROH_ for individuals with first cousin parents; Figure 2B).

To analyse the variance in F_ROH_ explained by confounders, we first residualised F_ROH_ using following linear model in each cohort (UKB EUR or UKB SAS) independently:

F_ROH_ ∼ sex + age + age^2^ + age^∗^sex + genetic_PCs1-20 + UKB_array + UKB_batch

We then assessed the variance explained (R^2^ and adjusted-R^2^) and model fit of the following regression:

residualised_F_ROH_ ∼ EduYears + ever_smoked + ever_alcohol + SES + religiosity + BMI + exercise + dietary_PC1 + dietary_PC2 + dietary_PC3 + dietary_PC4 + dietary_PC5

(EduYears: number of years in education; ever_smoked: ever smoked tobacco; ever_alcohol: ever drunk alcohol; SES: socioeconomic status as measured by Townsend Deprivation Index; religiosity: reported attending a religious group at least once a week; exercise: number of days/week of moderate physical activity for 10+ minutes; dietary_PC: principal component from a PCA of diet variables, described above under “Phenotype preparation for UK Biobank”)

We used G^∗^Power^29^ to calculate power to detect a significant model fit at p<0.05 or p<0.1 in a linear regression in the highly consanguineous cohorts, assuming that the predictors have the same R^2^ as that observed in the full cohorts, or half of this R^2^.

#### Analysis of Saudi Arabian dataset

We replicated the F_ROH_-T2D association in an independent cohort of 5,668 Saudi Arabian individuals that has been previously described.^66^ Phenotype data were extracted from medical records. Exclusion of relatives, PCA, ROH calling, and consanguinity inference were conducted in the same way as described above for the cohorts, resulting in 4,427 unrelated individuals, with 1,476 (33%) inferred to be offspring of first cousin/avuncular unions and F_ROH_ < 0.18. We conducted logistic regression, regressing T2D status on F_ROH_, genetic PCs 1-20, age, age^2^, sex, sex^∗^age in the full and highly consanguineous cohorts.

#### Within-sibling analysis in 23andMe

23andMe participants provided informed consent and volunteered to participate in the research online, under a protocol approved by the external AAHRPP-accredited IRB, Ethical & Independent (E&I) Review Services. As of 2022, E&I Review Services is part of Salus IRB (https://www.versiticlinicaltrials.org/salusirb).

We conducted a within-sibling regression analysis using individuals inferred to be full biological siblings in the 23andMe cohort, including individuals regardless of genetic ancestry since this within-family analysis is immune to population stratification. We considered 7,363,319 23andMe customers who had consented to research and had reported age, sex and at least one of the phenotypes of interest. Sibling groups were identified as cliques sharing 2249cM < IBD1 < 3373cM and 375cM < IBD2 < 2249cM.^75^ We then performed relatedness pruning to avoid (for example) two generations of a pedigree being analysed as independent sibling groups. For each phenotype, only cliques containing at least two individuals with non-missing data were considered, we then greedily removed cliques with the highest number of related cliques until no clique interconnections were remaining. Two cliques were considered connected if at least one pair across the cliques shared IBD1 > 700cM. This resulted in between 20,713 and 262,433 sibling cliques containing 42,218 to 545,806 individuals depending on phenotype. ROHs were called and F_ROH_ was determined in the same way as described above for G&H and UKB.

As 23andMe does not have electronic health records, we used self-reported phenotypes as proxies to replicate our significant findings from the meta-analysis of G&H and UKB. The results and lists of equivalent phenotypes are shown in Table S5. Using the *bife* R package^63^ to fit a fixed effect logistic regression model, we regressed the binary disease phenotype of interest on F_ROH_, adjusting for age, age^2^, sex, and sex^∗^age and family membership as a fixed effect (i.e. family-specific intercept). For quantitative phenotypes, we regressed the phenotype on the same covariates and family membership fixed effect using the *plm* R package.^65^ The analysis was conducted separately for three different genotyping chips, and the results meta-analysed.

To conduct a power calculation for each disease tested, we considered the number of discordant sibling pairs in 23andMe. At the time of completing this power calculation, we were not able to directly access the distribution of cases and controls across cliques, so we used a slightly conservative approach to appropriate sample sizes for power calculations across phenotypes. To derive an estimate of number sibling pairs to simulate, we sought to identify the number of unique intra-clique case-control pairs that were in the within-sibling analysis per phenotype. We first calculated the average clique size per phenotype, which was 2.03-2.08. We then calculated the number of cliques with n=2 siblings, which we call N_p_: N_p_ = round((average clique size - 1) ∗ # cases in discordant cliques)

N_p_ = round((average clique size - 1) ^∗^ # cases in discordant cliques)

We subsequently used this assumed number of sibling pairs in the simulation below. Intuitively, this approach accounts for the fact that in a clique with n=2, only one case-control comparison can be made, while in cliques with n=3, two unique case-control pairs exist.

We then simulated a dataset in the following way:

1. We first randomly chose an F_ROH_ value for the index sibling in a clique, which we call F_ROH,sib1_, sampling this from the F_ROH_ distribution of the UKB EUR full cohort (since most 23andMe participants have majority genetically-inferred European ancestries).
2. We then simulated the F_ROH_ value for their sibling from a normal distribution with mean = F_ROH,sib1_ and standard deviation = 0.002, the average within-sibling standard deviation of F_ROH_ values.
3. Using the F_ROH_ values, we simulated a binary phenotype with an odds ratio and prevalence equivalent to that estimated in the highly consanguineous cohorts from G&H+UKB.
4. We randomly selected N_p_ simulated sibling pairs that were discordant for case status, and fitted a fixed effect logistic regression model using the *bife* package (as described above) by regressing the phenotype on F_ROH_ with a family membership as a fixed effect.
5. We conducted 1,000 simulations for each disease, and calculated the power to detect an F_ROH_ association at a Bonferroni adjusted p-value (p<0.05/7).

To calculate the power to replicate at least one association, we used the following formula:

$$1-\sum_{i=1}^{n}\left(1-power\left(p_{i}\right)\right)$$

where $power\left(p_{i}\right)$ is the statistical power for phenotype $p_{i}$.

To calculate the power to replicate at least two associations, we used the formula:

$$1-\prod_{i=1}^{n}\left(1-power\left(p_{i}\right)\right)-\sum_{i=1}^{n}power\left(p_{i}\right)\prod_{j\neq i}\left(1-power\left(p_{j}\right)\right)$$

To conduct a power calculation for height, we considered the number of sibling cliques, N_c_, and simulated a dataset in the following way:

1. We first randomly chose an F_ROH_ value for the index sibling in a clique, as described above in the simulation of disease phenotypes.
2. We then simulated the F_ROH_ value for their sibling, as described above in the simulation of disease phenotypes.
3. Using the F_ROH_ values, we simulated a quantitative phenotype with effect size equivalent to that estimated in the highly consanguineous cohorts from G&H+UKB and normally distributed random error with mean 0 and standard deviation 9.4 (root of the variance of height minus the variance explained by F_ROH_).
4. We randomly selected N_c_ simulated sibling cliques, and fitted a linear mixed model model using the *plm* package (as described above) by regressing the simulated phenotype on F_ROH_ with a family membership as a fixed effect.

We conducted 1,000 simulations, and calculated the power to detect an F_ROH_ association at p = 0.05.

#### Calculating population attributable risk

To calculate population attributable risk for risk factor r (in our case, consanguinity) in population l as a percentage, we used the following formula:

$$PAR_{r,l}=100\times \frac{P_{r,l}\times \left(RR_{r}-1\right)}{P_{r,l}\times \left(RR_{r}-1\right)+1}$$

where $P_{r,l}$ is the prevalence of the risk factor r in population l and $RR_{r}$ is the risk ratio of the risk factor for the disease.

To convert the OR to RR, we used the following formula:

$$RR_{r}=\frac{OR_{r}}{1-P_{d,l}+P_{d,l}\times OR_{r}}$$

where $P_{d,l}$ is the prevalence of the disease in unexposed individuals (i.e. individuals with unrelated parents) and $OR_{r}$ is the odds ratio for a given level of autozygosity of a given disorder. As it is not possible at present to derive robust estimates of disease prevalence excluding individuals with related parents, we varied the disease prevalence from 5% to 15% for both diseases. In practice we found that this made little difference to our estimates (Figure 5). Since the calculation of PAR requires discrete risk factors, we discretized F_ROH_ into the values corresponding to the expectation for offspring of first cousins (F_ROH_ = 0.0625) and offspring of second cousins (F_ROH_ = 0.01562). We took the prevalence of these classes of consanguinity ($P_{r,l}$) from the estimates shown in Figure 1 for UKB EUR, G&H British Pakistanis and G&H British Bangladeshis separately.

Specifically, the PAR due to consanguinity for a given population, l, is given by:

$$PAR_{l}=100\times \left(\frac{P_{2,l}\times \left(RR_{0.0156}-1\right)}{P_{2,l}\times \left(RR_{0.0156}-1\right)+1}+\frac{P_{1,l}\times \left(RR_{0.0625}-1\right)}{P_{1,l}\times \left(RR_{0.0625}-1\right)+1}\right)$$

Where $RR_{F}$ is the risk ratio for having F_ROH_ = F, $P_{2,l}$ is the fraction of population l inferred to have parents who are second cousins/first cousins once removed, and $P_{1,l}$ the fraction inferred to have parents who are first cousins.

To calculate PAR for T2D attributable to a PRS, we used the OR estimates for a PRS developed in Mars et al.^36^ using the GWAS in Scott et al.,^76^ which they showed to have roughly equivalent degrees of predictive power in individuals with majority recent European versus South Asian ancestries. We used the same procedure as above, but calculated a risk ratio for individuals in twenty bins ranging from the top 1% to the top 20% of the PRS distribution, then calculated the cumulative sum of the PAR attributable to each 1% increment (Figure S4E).

#### Simulation of binary traits with strictly additive genetic architectures

We simulated the architecture of an additive binary trait by first assigning an effect size β drawn from N(0,1) for 1,000 independent causal loci. (We note that varying the parameter for the number of causal loci has no effect on our conclusions, as the genetic liability distribution for polygenic traits is normally distributed.) The allele frequency for each locus in the population was calculated by first calculating 1/β for each SNP and then linearly scaling the values to be between 0 and 0.5, to approximate model assumptions used in Schoech et al.^77^ (However, we note that the MAF-effect size relationship does not impact results as the additive variance and F_ROH_ relationship is not affected.) We then simulated 6,000 individuals (slightly more than the number of individuals in the combined highly consanguineous cohorts) to have F_ROH_ values either uniformly drawn from 0 to 1 or from the F_ROH_ distribution of the G&H highly consanguineous cohort (as shown in Figure 2B). For each individual, a random subset of round(1,000^∗^F_ROH_) SNPs were assigned to be autozygous. We then simulated genotypes for each individual with the genotype in non-autozygous segments drawn from Binomial(2,*p*) and from autozygous segments from 2^∗^Binomial(1,*p*) where *p* is the frequency of the effect allele at the locus. Genetic risk was then calculated by multiplying each individual’s genotypes with their corresponding effect sizes, summing them up, and then normalising the values across the cohort.

We then simulated a binary phenotype by drawing random values from Binomial(1,*p*_*d*_) where *p*_*d*_ is the probability an individual has the disease given their genetic risk score *G*, calculated as follows:

$$\mathrm{Pr}\left(disease\right)=\frac{L\left(G\;|\;N\left(d,1\right)\right)}{L\left(G\;\left|\;N\left(0,1\right))+L(G\;\right|\;N\left(d,1\right)\right)}$$

where L(*G* | *D*) is the probability density of genetic risk score *G* with respect to a distribution *D* and *d* is the mean shift in the distribution of genetic risk among cases, ranging from 0.5-1.5 in increments of 0.1. The heritability was calculated using Nagelkerke pseudo-R^2^ in a logistic regression of the phenotype on *G*. We then carried out a logistic regression of simulated phenotype on F_ROH_. We repeated this for 100 simulations, and then calculated power as the fraction of simulations in which the F_ROH_ effect size was positive and its p-value was less than a given cutoff (p<0.05 or p<0.05/61).

## Data Availability

• G&H data are available for analysis within a secure Trusted Research Environment) upon application to the G&H executive, as described here https://www.genesandhealth.org/research/scientists-using-genes-health-scientific-research. UK Biobank data are also available upon application (https://www.ukbiobank.ac.uk/enable-your-research/apply-for-access). The informed consent given by the Saudi study participants does not allow posting of participant-level phenotype and genotype data in public databases. Access to these data can be obtained through an established ISO-certified process by submitting a project request to the Office of Research Affairs, King Faisal Specialist Hospital & Research Centre (KFSHRC) (ORA@kfshrc.edu.sa) which is subject to approval by the KFSHRC IRB committee. The summary statistics from the sibling analysis for the 23andMe replication dataset are fully disclosed in the manuscript. Individual-level data are not publicly available due participant confidentiality, and in accordance with the IRB-approved protocol under which the study was conducted. • All original code is available upon request. The code to infer consanguinity is available at https://github.com/malawsky/consanguinity_simulation. • Any additional information required to reanalyse the data reported in this paper is available from the lead contact upon request.

## Supplemental information

Document S1. Note S1

Table S1. Variance in F_ROH_ explained by potential confounders (EduYears, ever_smoked, ever_alcohol, SES, religiosity, BMI, exercise, and five dietary PCs) the full cohorts and highly consanguineous cohorts from UKB EUR and UKB SAS, related to Figure 3 R^2^ values and model fit p-value are reported for a regression of F_ROH_ on these confounders, after regressing out standard covariates (see STAR Methods). Power to detect significant model fits in each of the highly consanguineous UKB cohorts based on the R^2^ values in the respective full cohorts is reported.

Table S2. Associations between F_ROH_ and height in the full, highly consanguineous cohorts from G&H and UKB and within-sibling 23andMe cohort, related to Figure 4 and Tables S3 and S5 Columns indicate the effect sizes (beta), standard errors (SE), and p-values for the associations.

Table S3. Associations between F_ROH_ and ICD10 codes in the highly consanguineous and full cohorts from G&H and UKB, related to Figures 4 and S3 Columns indicate the effect sizes (beta), standard errors (SE), and p-values for the associations in each cohort as well as in the meta-analysis. FDR corrected q-values and Cochrane’s Q test for heterogeneity are shown for the meta-analysis.

Table S4. Sensitivity analyses of associations between F_ROH_ and ICD10 codes that were significant (FDR < 5%) in the analysis of the highly consanguineous cohorts, related to Figures 4 and S3 Columns A–N: associations between F_ROH_ and ICD10 codes in the highly consanguineous cohorts from UKB SAS, UKB EUR and G&H, resulting from meta-analysis of three separate regressions stratified by number of generations of inferred first cousin parental relatedness (PR-stratified meta-analysis) and then meta-analysed across the three cohorts. Column O–Z: associations between F_ROH_ and ICD10 codes in the highly consanguineous cohorts from UKB SAS and UKB EUR highly consanguineous cohorts without and with the addition of confounders included in the regression (EduYears, ever_smoked, ever_alcohol, SES, religiosity, BMI, exercise, and five dietary PCs). Column AA-AL: associations between F_GRM_ and ICD10 in the highly consanguineous cohorts. Columns indicate the effect sizes (beta), standard errors (SE), and p-values for the associations in each cohort as well as in the meta-analysis.

Table S5. Associations between F_ROH_ and phenotypes in the within-sibling analysis from 23andMe, related to Figure 4 We show the effect size (beta), standard error (SE), p value from a meta-analysis across three chips, as well as the various summaries of the sample size and an estimate of the power for replication from simulations.

Document S2. Frequently asked questions A document of “frequently asked questions” regarding this paper, aimed at a lay audience.

## References

[1] Bittles A.H., Black M.L. (2010). Evolution in health and medicine Sackler colloquium: consanguinity, human evolution, and complex diseases. Proc. Natl. Acad. Sci. USA.

[2] Arciero E., Dogra S.A., Malawsky D.S., Mezzavilla M., Tsismentzoglou T., Huang Q.Q., Hunt K.A., Mason D., Sharif S.M., van Heel D.A. (2021). Fine-scale population structure and demographic history of British Pakistanis. Nat. Commun..

[3] Basu A., Mukherjee N., Roy S., SenGupta S., Banerjee S., Chakraborty M., Dey B., Roy M., Roy B., Bhattacharyya N.P. (2003). Ethnic India: a genomic view, with special reference to peopling and structure. Genome Res..

[4] Reich D., Thangaraj K., Patterson N., Price A.L., Singh L. (2009). Reconstructing Indian population history. Nature.

[5] Small N., Bittles A.H., Petherick E.S., Wright J. (2017). Endogamy, consanguinity and the health implications of changing marital choices in the UK Pakistani community. J. Biosoc. Sci..

[6] Ceballos F.C., Joshi P.K., Clark D.W., Ramsay M., Wilson J.F. (2018). Runs of homozygosity: windows into population history and trait architecture. Nat. Rev. Genet..

[7] Sheridan E., Wright J., Small N., Corry P.C., Oddie S., Whibley C., Petherick E.S., Malik T., Pawson N., McKinney P.A. (2013). Risk factors for congenital anomaly in a multiethnic birth cohort: an analysis of the Born in Bradford study. Lancet.

[8] Martin H.C., Jones W.D., McIntyre R., Sanchez-Andrade G., Sanderson M., Stephenson J.D., Jones C.P., Handsaker J., Gallone G., Bruntraeger M. (2018). Quantifying the contribution of recessive coding variation to developmental disorders. Science.

[9] Clark D.W., Okada Y., Moore K.H.S., Mason D., Pirastu N., Gandin I., Mattsson H., Barnes C.L.K., Lin K., Zhao J.H. (2019). Associations of autozygosity with a broad range of human phenotypes. Nat. Commun..

[10] Johnson E.C., Evans L.M., Keller M.C. (2018). Relationships between estimated autozygosity and complex traits in the UK Biobank. PLoS Genet..

[11] Napolioni V., Scelsi M.A., Khan R.R., Altmann A., Greicius M.D. (2020). Recent consanguinity and outbred autozygosity are associated with increased risk of late-onset Alzheimer’s disease. Front. Genet..

[12] Christofidou P., Nelson C.P., Nikpay M., Qu L., Li M., Loley C., Debiec R., Braund P.S., Denniff M., Charchar F.J. (2015). Runs of homozygosity: association with coronary artery disease and gene expression in monocytes and macrophages. Am. J. Hum. Genet..

[13] Barnett A.H., Dixon A.N., Bellary S., Hanif M.W., O’Hare J.P., Raymond N.T., Kumar S. (2006). Type 2 diabetes and cardiovascular risk in the UK south Asian community. Diabetologia.

[14] Bellary S., Barnett A. (2007). Diabetes and CVD in South Asians: a review. Pediatr Diab..

[15] Srinivasan S., Liju S., Sathish N., Siddiqui M.K., Anjana R.M., Pearson E.R., Doney A.S.F., Mohan V., Radha V., Palmer C.N.A. (2022). Common and distinct genetic architecture of age at diagnosis of diabetes in south Indian and European populations. bioRxiv.

[16] Charlesworth D., Willis J.H. (2009). The genetics of inbreeding depression. Nat. Rev. Genet..

[17] Falconer D.S. (1995).

[18] Keller M.C., Simonson M.A., Ripke S., Neale B.M., Gejman P.V., Howrigan D.P., Lee S.H., Lencz T., Levinson D.F., Sullivan P.F. (2012). Runs of homozygosity implicate autozygosity as a schizophrenia risk factor. PLoS Genet..

[19] Heron E.A., Cormican P., Donohoe G., O’Neill F.A., Kendler K.S., Riley B.P., Gill M., Corvin A.P., Morris D.W., Wellcome Trust Case Control Consortium 2 (2014). No evidence that runs of homozygosity are associated with schizophrenia in an Irish genome-wide association dataset. Schizophr. Res..

[20] Johnson E.C., Bjelland D.W., Howrigan D.P., Abdellaoui A., Breen G., Borglum A., Cichon S., Degenhardt F., Forstner A.J., Frank J. (2016). No reliable association between runs of homozygosity and schizophrenia in a well-powered replication study. PLOS Genet..

[21] Abdellaoui A., Hottenga J.-J., Xiao X., Scheet P., Ehli E.A., Davies G.E., Hudziak J.J., Smit D.J.A., Bartels M., Willemsen G. (2013). Association between autozygosity and major depression: stratification due to religious assortment. Behav. Genet..

[22] Saccheri I.J., Lloyd H.D., Helyar S.J., Brakefield P.M. (2005). Inbreeding uncovers fundamental differences in the genetic load affecting male and female fertility in a butterfly. Proc. Biol. Sci..

[23] Sved J.A. (1971). An estimate of heterosis in Drosophila melanogaster. Genet. Res..

[24] Latter B.D., Mulley J.C., Reid D., Pascoe L. (1995). Reduced genetic load revealed by slow inbreeding in *Drosophila melanogaster*. Genetics.

[25] Schrieber K., Paul S.C., Höche L.V., Salas A.C., Didszun R., Mößnang J., Müller C., Erfmeier A., Eilers E.J. (2021). Inbreeding in a dioecious plant has sex- and population origin-specific effects on its interactions with pollinators. eLife.

[26] Thornhill N.W. (1993).

[27] Finer S., Martin H.C., Khan A., Hunt K.A., MacLaughlin B., Ahmed Z., Ashcroft R., Durham C., MacArthur D.G., McCarthy M.I. (2020). Cohort Profile: East London Genes & Health (ELGH), a community-based population genomics and health study in British Bangladeshi and British Pakistani people. Int. J. Epidemiol..

[28] Colbert S.M.C., Wendt F.R., Pathak G.A., Helmer D.A., Hauser E.R., Keller M.C., Polimanti R., Johnson E.C. (2023). Declining autozygosity over time: an exploration in over 1 million individuals from three diverse cohorts. Am. J. Hum. Genet..

[29] Faul F., Erdfelder E., Lang A.-G., Buchner A. (2007). G^∗^Power 3: a flexible statistical power analysis program for the social, behavioral, and biomedical sciences. Behav. Res. Methods.

[30] Ceballos F.C., Hazelhurst S., Clark D.W., Agongo G., Asiki G., Boua P.R., Xavier Gómez-Olivé F., Mashinya F., Norris S., Wilson J.F. (2020). Autozygosity influences cardiometabolic disease-associated traits in the AWI-Gen sub-Saharan African study. Nat. Commun..

[31] Howe L.J., Nivard M.G., Morris T.T., Hansen A.F., Rasheed H., Cho Y., Chittoor G., Ahlskog R., Lind P.A., Palviainen T. (2022). Within-sibship genome-wide association analyses decrease bias in estimates of direct genetic effects. Nat. Genet..

[32] Young A.I., Benonisdottir S., Przeworski M., Kong A. (2019). Deconstructing the sources of genotype-phenotype associations in humans. Science.

[33] Sheikh A., Steiner M.F., Cezard G., Bansal N., Fischbacher C., Simpson C.R., Douglas A., Bhopal R., SHELS researchers (2016). Ethnic variations in asthma hospital admission, readmission and death: a retrospective, national cohort study of 4.62 million people in Scotland. BMC Med..

[34] Goff L.M. (2019). Ethnicity and Type 2 diabetes in the UK. Diabet. Med..

[35] Netuveli G., Hurwitz B., Levy M., Fletcher M., Barnes G., Durham S.R., Sheikh A. (2005). Ethnic variations in UK asthma frequency, morbidity, and health-service use: a systematic review and meta-analysis. Lancet.

[36] Mars N., Kerminen S., Feng Y.-C.A., Kanai M., Läll K., Thomas L.F., Skogholt A.H., Della Briotta Parolo P., FinnGen, Biobank Japan Project (2022). Genome-wide risk prediction of common diseases across ancestries in one million people. Cell Genom..

[37] Xue A., Wu Y., Zhu Z., Zhang F., Kemper K.E., Zheng Z., Yengo L., Lloyd-Jones L.R., Sidorenko J., Wu Y. (2018). Genome-wide association analyses identify 143 risk variants and putative regulatory mechanisms for type 2 diabetes. Nat. Commun..

[38] Bittles A.H., Mason W.M., Greene J., Rao N.A. (1991). Reproductive behavior and health in consanguineous marriages. Science.

[39] Bittles A.H., Speicher M.R., Motulsky A.G., Antonarakis S.E. (2010). Vogel and Motulsky’s Human Genetics.

[40] Shaw A. (2014). Drivers of cousin marriage among British Pakistanis. Hum. Hered..

[41] Hamamy H. (2012). Consanguineous marriages: preconception consultation in primary health care settings. J. Community Genet..

[42] Hsu C.L., Sheu W.H.-H. (2016). Diabetes and shoulder disorders. J. Diabetes Investig..

[43] Roberts A.L., Agnew-Blais J.C., Spiegelman D., Kubzansky L.D., Mason S.M., Galea S., Hu F.B., Rich-Edwards J.W., Koenen K.C. (2015). Posttraumatic stress disorder and incidence of type 2 diabetes mellitus in a sample of women: a 22-year longitudinal study. JAMA Psychiatry.

[44] Bhopal R.S. (2013). A four-stage model explaining the higher risk of type 2 diabetes mellitus in South Asians compared with European populations. Diabet. Med..

[45] Nightingale C.M., Rudnicka A.R., Kerry-Barnard S.R., Donin A.S., Brage S., Westgate K.L., Ekelund U., Cook D.G., Owen C.G., Whincup P.H. (2018). The contribution of physical fitness to individual and ethnic differences in risk markers for type 2 diabetes in children: the Child Heart and Health Study in England (CHASE). Pediatr. Diabetes.

[46] Owen C.G., Nightingale C.M., Rudnicka A.R., Cook D.G., Ekelund U., Whincup P.H. (2009). Ethnic and gender differences in physical activity levels among 9–10-year-old children of white European, South Asian and African-Caribbean origin: the Child Heart Health Study in England (CHASE Study). Int. J. Epidemiol..

[47] Donin A.S., Nightingale C.M., Owen C.G., Rudnicka A.R., McNamara M.C., Prynne C.J., Stephen A.M., Cook D.G., Whincup P.H. (2010). Nutritional composition of the diets of South Asian, black African-Caribbean and white European children in the United Kingdom: the Child Heart and Health Study in England (CHASE). Br. J. Nutr..

[48] Bryant M., Sahota P., Santorelli G., Hill A. (2015). An exploration and comparison of food and drink availability in homes in a sample of families of White and Pakistani origin within the UK. Public Health Nutr..

[49] Yates T., Davies M.J., Gray L.J., Webb D., Henson J., Gill J.M.R., Sattar N., Khunti K. (2010). Levels of physical activity and relationship with markers of diabetes and cardiovascular disease risk in 5474 white European and South Asian adults screened for type 2 diabetes. Prev. Med..

[50] Harnett N.G., Ressler K.J. (2021). Structural racism as a proximal cause for race-related differences in psychiatric disorders. Am. J. Psychiatry.

[51] Lillis T.A., Burns J., Aranda F., Purim-Shem-Tov Y.A., Bruehl S., Beckham J.C., Hobfoll S.E. (2018). PTSD symptoms and acute pain in the emergency department: the roles of vulnerability and resilience factors among low-income, inner-city women. Clin. J. Pain.

[52] GBD (2022). Burden of diabetes and hyperglycaemia in adults in the Americas, 1990–2019: a systematic analysis for the Global Burden of Disease Study 2019. Lancet Diabetes Endocrinol..

[53] Dehghan A., van Hoek M., Sijbrands E.J.G., Stijnen T., Hofman A., Witteman J.C.M. (2007). Risk of type 2 diabetes attributable to C-reactive protein and other risk factors. Diabetes Care.

[54] Heyne H.O., Karjalainen J., Karczewski K.J., Lemmelä S.M., Zhou W., Gen F., Havulinna A.S., Kurki M., Rehm H.L., Palotie A. (2021).

[55] O’Connor M.J., Schroeder P., Huerta-Chagoya A., Cortés-Sánchez P., Bonàs-Guarch S., Guindo-Martínez M., Cole J.B., Kaur V., Torrents D., Veerapen K. (2022). Recessive genome-Wide Meta-analysis illuminates genetic architecture of Type 2 diabetes. Diabetes.

[56] Guindo-Martínez M., Amela R., Bonàs-Guarch S., Puiggròs M., Salvoro C., Miguel-Escalada I., Carey C.E., Cole J.B., Rüeger S., Atkinson E. (2021). The impact of non-additive genetic associations on age-related complex diseases. Nat. Commun..

[57] Palmer D.S., Zhou W., Abbott L., Baya N., Churchhouse C., Seed C., Poterba T., King D., Kanai M., Bloemendal A. (2022).

[58] Hivert V., Sidorenko J., Rohart F., Goddard M.E., Yang J., Wray N.R., Yengo L., Visscher P.M. (2021). Estimation of non-additive genetic variance in human complex traits from a large sample of unrelated individuals. Am. J. Hum. Genet..

[59] Yengo L., Wray N.R., Visscher P.M. (2019). Extreme inbreeding in a European ancestry sample from the contemporary UK population. Nat. Commun..

[60] Curik I., Sölkner J., Stipic N. (2001). The influence of selection and epistasis on inbreeding depression estimates. J. Anim. Breed. Genet..

[61] Fairley S., Lowy-Gallego E., Perry E., Flicek P. (2020). The International Genome Sample Resource (IGSR) collection of open human genomic variation resources. Nucleic Acids Res..

[62] Bycroft C., Freeman C., Petkova D., Band G., Elliott L.T., Sharp K., Motyer A., Vukcevic D., Delaneau O., O’Connell J. (2018). The UK biobank resource with deep phenotyping and genomic data. Nature.

[63] Stammann A., Heiss F., McFadden D. (2016). https://cran.r-project.org/web/packages/bife/index.html.

[64] Manichaikul A., Mychaleckyj J.C., Rich S.S., Daly K., Sale M., Chen W.-M. (2010). Robust relationship inference in genome-wide association studies. Bioinformatics.

[65] Croissant Y., Millo G. (2008). Panel Data Econometrics inR: TheplmPackage. J. Stat. Softw..

[66] Wakil S.M., Ram R., Muiya N.P., Mehta M., Andres E., Mazhar N., Baz B., Hagos S., Alshahid M., Meyer B.F. (2016). A genome-wide association study reveals susceptibility loci for myocardial infarction/coronary artery disease in Saudi Arabs. Atherosclerosis.

[67] Huang Q.Q., Sallah N., Dunca D., Trivedi B., Hunt K.A., Hodgson S., Lambert S.A., Arciero E., Wright J., Griffiths C. (2022). Transferability of genetic loci and polygenic scores for cardiometabolic traits in British Pakistani and Bangladeshi individuals. Nat. Commun..

[68] Loh P.R., Danecek P., Palamara P.F., Fuchsberger C., A Reshef Y., K Finucane H., Schoenherr S., Forer L., McCarthy S., Abecasis G.R. (2016). Reference-based phasing using the Haplotype Reference Consortium panel. Nat. Genet..

[69] Lawson D.J., Hellenthal G., Myers S., Falush D. (2012). Inference of population structure using dense haplotype data. PLoS Genet..

[70] Meyer H.V. (2020).

[71] Venables W.N., Ripley B.D. (2002).

[72] NIH (2012). https://www.nlm.nih.gov/research/umls/mapping_projects/snomedct_to_icd10cm.html.

[73] Okbay A., Wu Y., Wang N., Jayashankar H., Bennett M., Nehzati S.M., Sidorenko J., Kweon H., Goldman G., Gjorgjieva T. (2022). Polygenic prediction of educational attainment within and between families from genome-wide association analyses in 3 million individuals. Nat. Genet..

[74] Cole J.B., Florez J.C., Hirschhorn J.N. (2020). Comprehensive genomic analysis of dietary habits in UK Biobank identifies hundreds of genetic associations. Nat. Commun..

[75] Henn B.M., Hon L., Macpherson J.M., Eriksson N., Saxonov S., Pe’er I., Mountain J.L. (2012). Cryptic distant relatives are common in both isolated and cosmopolitan genetic samples. PLoS One.

[76] Scott R.A., Scott L.J., Mägi R., Marullo L., Gaulton K.J., Kaakinen M., Pervjakova N., Pers T.H., Johnson A.D., Eicher J.D. (2017). An expanded genome-wide association study of Type 2 diabetes in Europeans. Diabetes.

[77] Schoech A.P., Jordan D.M., Loh P.-R., Gazal S., O’Connor L.J., Balick D.J., Palamara P.F., Finucane H.K., Sunyaev S.R., Price A.L. (2019). Quantification of frequency-dependent genetic architectures in 25 UK Biobank traits reveals action of negative selection. Nat. Commun..

