# Supplementary material for: Influence of autozygosity on common disease risk across the phenotypic spectrum
Source: Cell. 2023 Oct 12;186(21):4514–4527.e14. doi: 10.1016/j.cell.2023.08.028 (PMC10580289; doi:10.1016/j.cell.2023.08.028)
Supplement: Document S1. Note S1 [file mmc1.pdf]

Cell, Volume *186*

Supplemental information

Influence of autozygosity on common disease risk across the phenotypic spectrum

Daniel S. Malawsky, Eva van Walree, Benjamin M. Jacobs, Teng Hiang Heng, Qin Qin Huang, Ataf H. Sabir, Saadia Rahman, Saghira Malik Sharif, Ahsan Khan, Maša Umićević Mirkov, 23andMe Research Team, Genes & Health Research Team, Hiroyuki Kuwahara, Xin Gao, Fowzan S. Alkuraya, Danielle Posthuma, William G. Newman, Christopher J. Griffiths, Rohini Mathur, David A. van Heel, Sarah Finer, Jared O'Connell, and Hilary C. Martin

Explanation for how autozygosity influences the additive variance of a trait

For illustration, consider a causal locus for a genetically additive trait as is assumed in standard GWAS, where being heterozygous increases risk towards the disease and being homozygous for the alternate allele increases risk twice as much as being heterozygous. Thus, we can code the risk incurred at the locus as 0 for homozygous reference allele, 1 for heterozygous, and 2 for homozygous alternate allele. Assume the locus has risk allele frequency *p*. For an individual not autozygous at the locus, the variance for the coded genotype is equivalent to the variance of a binomial distribution Var(f_1) = σ_1^2 = Var(Binomial(2,p)) = 2p(1-p). However, for an individual autozygous at the locus, the variance is equivalent to Var(f_2) = σ_2^2 = Var(2*Binomial(1,p)) = 4p(1-p).

Given an individual with inbreeding coefficient F (which we approximate by F_{ROH}), the variance at the locus of the mixture distribution is:

$$Var((1-F)\times f_1 + F\times f_2) =$$
$$(1-F)\times \sigma_1^2 + F\times \sigma_2^2 =$$
$$(1-F)\times \sigma_1^2 + 2F\times \sigma_1^2 =$$
$$(1+F)\times \sigma_1^2$$

which is equivalent to results of a complementary derivation from Falconer et al. [S1]

Thus, extending this argument to multiple risk loci, autozygosity linearly increases variance in risk towards a trait that has an entirely additive architecture. Assuming a liability threshold model, the increased additive variance will lead to individuals with higher F_{ROH} having a greater chance of passing the disease threshold even in the absence of non-additive effects, and may induce an association between F_{ROH} and the trait.

References

1. Falconer, D.S. (1995). Introduction to Quantitative Genetics (4th Edition) 4th ed. (Longman).
